# Tribological Performance of High-Entropy Coatings (HECs): A Review

**DOI:** 10.3390/ma15103699

**Published:** 2022-05-21

**Authors:** Payank Patel, Amit Roy, Navid Sharifi, Pantcho Stoyanov, Richard R. Chromik, Christian Moreau

**Affiliations:** 1 Department of Mechanical, Industrial and Aerospace Engineering, Concordia University, Montreal, QC H3G 1M8, Canada; amit.roy@concordia.ca (A.R.); navid.sharifi@concordia.ca (N.S.); 2 Department of Mining and Materials Engineering, McGill University, Montreal, QC H3A 0G4, Canada; 3 Department of Chemical and Materials Engineering, Concordia University, Montreal, QC H3G 1M8, Canada

**Keywords:** high-entropy coatings (HECs), high-entropy alloys (HEAs), configurational, solid solutions, feedstock, tribological behaviour

## Abstract

Surface coatings that operate effectively at elevated temperatures provide compatibility with critical service conditions as well as improved tribological performance of the components. High-entropy coatings (HECs), including metallic, ceramics, and composites, have gained attention all over the world and developed rapidly over the past 18 years, due to their excellent mechanical and tribological properties. High-entropy alloys (HEAs) are defined as alloys containing five or more principal elements in equal or close to equal atomic percentage. Owing to the high configurational entropy compared to conventional alloys, HEAs are usually composed of a simple solid solution phase, such as the BCC and FCC phases, instead of complex, brittle intermetallic phases. Several researchers have investigated the mechanical, oxidation, corrosion and wear properties of high-entropy oxides, carbides, borides, and silicates using various coating and testing techniques. More recently, the friction and wear characteristics of high-entropy coatings (HECs) have gained interest within various industrial sectors, mainly due to their favourable mechanical and tribological properties at high temperatures. In this review article, the authors identified the research studies and developments in high-entropy coatings (HECs) fabricated on various substrate materials using different synthesis methods. In addition, the current understanding of the HECs characteristics is critically reviewed, including the fabrication routes of targets/feedstock, synthesis methods utilized in various research studies, microstructural and tribological behaviour from room temperature to high temperatures.

## 1. Introduction

The term tribology is not well recognized among the wider population and can be frequently misunderstood. The science and technology of interacting surfaces in relative motion is known as tribology. It includes the principles and applications of friction, wear, and lubrication [1]. For example, from shaving, where cream is used as a lubricant to minimize friction between the razor and the skin, to advanced technical applications, where optimum levels of friction, wear, and lubrication are required to ensure adequate performance [2]. A typical tribological system is a combination of four components [3]. The first two elements are represented by the surfaces of the two bodies sliding against each other. The third element is an interfacial medium, which can be a lubricant (solid, liquid, gas, or a combination of these) or the other variety of intermediate layer. The final element is the surrounding environment. Wear and frictional behaviour are not inherent properties of the material, but rather a result of the aforementioned factors, their properties, and interactions over time. With the advancement of science and technology, extreme tribology or high temperature tribology has gained significant interest in various sectors, such as aerospace (air foil bearing, rolling element bearing, hot and cold section of the engine, satellite components), automobile (engine bearings, piston, traction drive), metal forming, and energy generation industries [4,5,6].

**Figure 1:**
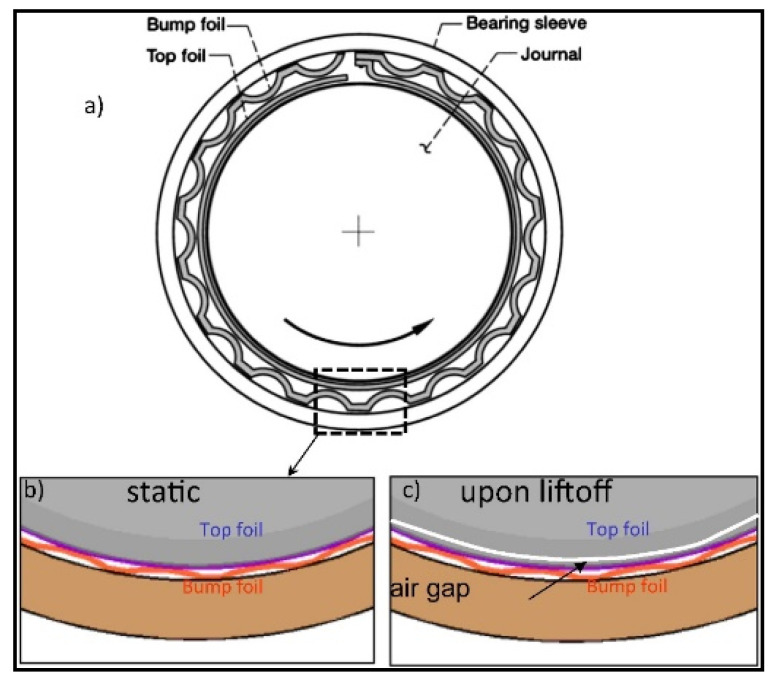
Schematic of an air foil bearing (**a**). Figures (**b**,**c**) shows an enlarged view of the journal/top foil interface while the bearing is static and dynamic or upon lift-off respectively [10].

Air foil bearings are one example of a component used at high temperatures that does not require liquid lubrication. As illustrated in Figure 1, the system consists of a top and a bump foil that support the rotating shaft. When stationary, the foil makes contact with the shaft, and at greater velocities, hydrodynamic pressure creates a thin film/gap between the top foil and the journal [7]. Thus, at low velocities, such as during start-up acceleration (room temperature) and shutdown deceleration (high temperature), the bearing touches the race. The variability of these working circumstances causes challenges since the materials must deliver lubricity throughout a wide temperature range for an extended period of time. The rolling element bearing of a gas turbine engine, as well as the bearing of an automotive diesel engine and its piston, work at high temperatures in same way that air foil bearings do. [8]. Similarly, the next-generation gas turbine engines must significantly lower fuel consumption, which necessitates a significant change in the design and operating environment of the mechanical systems (e.g., higher temperatures, increased velocities, and contact pressures) [9]. 

**Figure 2:**
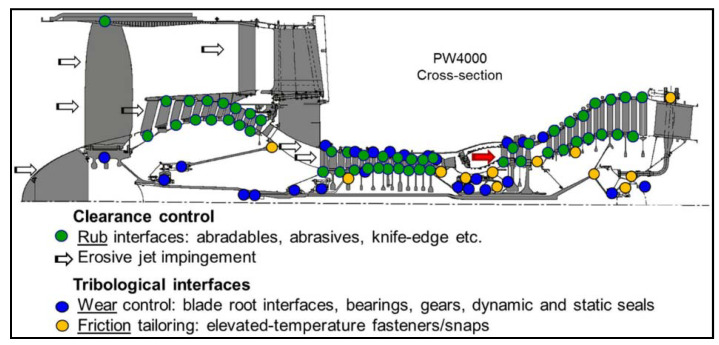
Example of contact locations in a jet engine [9].

Figure 2 depicts a complete cross-section of a jet engine, with contacting interfaces categorized into two types: (i) clearance control interfaces and (ii) tribological interfaces. The performance of the engine is governed by the tied clearances; A 1% reduction in engine bleed results in a 0.4 percent reduction in specific fuel consumption [11]. Thus, materials with optimal performance for clearance control can significantly improve the efficiency of the engine. Similarly, in aerospace industries, the multiple complicated contacting and moving mechanical components in a gas turbine engine can limit the lifespan of the specific structures [9,12]. However, the components of gas turbine engines are expected to exhibit adequate durability, precision, and high structure stability in order to enhance the operational safety. 

One component in the aeroengine that require significant consideration of surface engineering and tribology in its design are static seals. They are used in multiple sections across gas turbine engines and thus, static seals exist in a variety of configurations with a range of temperature requirements. Static seals are thin sheet metal gaskets, located between various engine components, with the primary purpose of preventing hot gases from leaking out into the cold area. Ni- and Co--based alloys have traditionally been the primary choice of material for metallic sealing applications in the hot sections of the engine, mainly due to the temperature capability and ease of formability. Nickel is easy to form into sheet metal and subsequently the gasket needed [13]. However, due to their poor tribological performance (i.e., high friction and wear), current static seal materials are not capable of effectively operating under the harsh conditions and can result in rapid wear and seal failure. Periodic replacement, which can become expensive, is required for the seals. Also, if seal damage is extensive before a repair or replacement can take place, damage can occur to the more expensive material for which the seal is in contact (i.e., the “counterface”). Overall, reliable seals with longer lifetime have significant benefits for the safety, maintainability and costs associated with aeroengines. Also, it should be noted that effective sealing is necessary to obtain the desired turbine efficiency and output. Without high quality seals, the aeroengine is less efficient and burns more fuel. Another example is friction brakes in a railway disc assembly, where the thermal phenomena is extremely important [14]. Vehicle deceleration and stopping are entirely dependent on friction (sliding contact), and the process must be predictable and reliable in order to ensure safe operation. It should be mentioned that, in addition to significant mechanical forces, friction heat generation is extremely high. In heavy duty braking applications, the heat flux at the contact is on the order of MW/m^2^. Heat generated during braking produces an increase in temperature at the interface, which quickly spreads through the brake components. Such severe thermal processes alter the friction properties of the materials in contact, causing wear and, on a larger scale, component deflection. All these alterations will inevitably have an impact on brake performance and life [14]. 

Furthermore, the cutting and machining tools which are being used in the machine industries need to operate under harsh conditions including elevated temperatures or under high loads with the minimum use of lubricants. Such metal working processes can exhibit temperatures of around 800–1200 °C [15], which can cause degradation of the workpiece and consequently lead to premature failure at high temperatures. Therefore, the friction (i.e., leading to frictional heating) between metal and forming tools has a great influence on process performance and finished products [16]. Besides, the number of applications and technological processes operating under harsh conditions like high temperatures has increased in recent years. To enable the development of new products and methods intended for such situations, there is a need for new knowledge about tribological phenomena occurring at high temperatures and related solutions. There is a particular interest in the friction and wear characteristics of high-entropy coatings (HECs) since they have been introduced and can also be employed in many other industrial applications, especially at high temperatures. Owing to the superior combination of mechanical and thermal properties, high-entropy alloys (HEAs) and coatings (HECs) gained attention globally. However, their tribological behaviour as the material of choice under harsh environments is still unknown. Even though several studies have been conducted to bridge this knowledge gaps, high temperature tribology has not received adequate attention 

## 2. High-Entropy Alloys (HEAs) and Coatings (HECs)

Over the past decades, most alloys have been developed with a single principal element by incorporating a variety of different alloy elements to improve overall mechanical and physical properties based on requirements [17]. However, the first study was carried out in the year 2004 by Yeh et al. [18], and Cantor et al. [19] and Ranganathan et al. [20] introduced the concept of high-entropy alloys (HEAs) consisting of five or more principal elements with each element concentration varying from 5 to 35 at.% [20,21,22,23,24,25,26,27,28,29,30]. Depending on the alloy compositions and temperature, HEAs can easily form simple solid solution phases, such as the BCC, B2, and FCC phases, instead of complex, brittle intermetallic phases [20,31]. These solid solution phases are mainly attributed to high entropy mixing (ΔS_mix_), which can be explained by Gibbs’s free energy equilibrium, which can be represented in Equation (1) [21,27,32,33,34]. However, it is worth mention that the term ΔS_mix_ includes all entropy sources such as configurational, vibrational, electronic, and magnetic contributions.

(1) $${\Delta G}_{\mathrm{mix}}={\Delta H}_{\mathrm{mix}}-T\Delta S$$

where ΔH_mix_: enthalpy of mixing, T: temperature, and ΔS_mix_: entropy of mixing.

For equiatomic high-entropy alloys, configurational entropy (ΔS_conf_) can be expressed by Equation (2) [27,33,35,36]:

(2) $${\Delta S}_{\mathrm{conf}}=-\mathrm{Rln}\frac{1}{n}=\;\mathrm{Rln}\;n$$

where R: gas constant, and n: number of elements.

Based on the configurational entropy (ΔS_conf_), there are a total of three categories (low, medium, and high entropy) for alloys; if the ΔSconf is less than 1R, then low-entropy alloys, also known as conventional alloys, and 1R ≤ ΔS_conf_ ≤ 1.6R are medium-entropy alloys. However, to produce high-entropy alloys, ΔS_conf_ must be maintained at more than 1.6R. The configurational entropies of compositionally complex alloys are high in the liquid or fully random solid solution state. To avoid further confusions, a sort of arbitrary threshold of configurational entropy larger than 1.6R (where R is the gas constant) was proposed as an operational definition for HEAs [36]. The main reason behind the novel behaviour of HEAs is the four different core mechanisms/effects. These effects could enhance the overall mechanical and metallurgical properties of HEAs. Many authors [26,27,37,38] have suggested four core effects and described the influence on the properties of HEAs: (1) high entropy effects, (2) severe lattice distortion, (3) sluggish diffusion, and (4) cocktail effects [20,39]. Based on the empirical rules, HEAs tend to form single solid solutions under the following conditions. (i) The valence electron concentration (VEC) is equal to or greater than 8 [40]; (ii) a new parameter combining effect of entropy and enthalpy (Ω) was proposed to be more than 1 and atomic size difference (δ) less than 6.5% [41,42]. Yang et al. [29] calculated and reported these parameters for various HEA systems. The parameters VEC, Ω, and δ are defined as the following equations, respectively [41].

(3) $$VEC=\sum_{i=0}^{n}X_{i}\;{\left(VEC\right)}_{i}$$

where (*VEC*) *i* is valence electron concentration of the *i*th component, *X_i_* is the mole fraction of the *i*th component.

(4) $$\Omega =\frac{T_{m}\;\Delta S_{mix}}{\left|\Delta H_{mix}\right|}$$

where *T_m_* is the melting temperature of the system, calculated as the average of melting temperatures of its components. R is the gas constant, 8.314 J/K. mol., Δ*S_mix_* the configurational entropy.

(5) $$\delta =\sqrt{\sum_{i=0}^{n}X_{i}\;\left(1-r_{i}/\bar{r}\right){\;}^{2}}$$

where *r_i_* is the atomic radius of *i*th component, and *r* is the mean atomic radius of all elements.

Although the concept of “HEAs” was initially applied to alloy design, it was rapidly extended to other materials such as high-entropy ceramics, composites, and polymers, significantly expanding the diversity of such high-entropy material systems. These are all examples of high-entropy materials (HEMs). “High-entropy coatings” (HECs), which include high-entropy metallic, ceramic, and composite coatings, are one of the most important research areas of HEMs, with articles on the subject first appearing briefly in 2004, just after the HEAs concept was established. Since then, many authors have studied several HEAs and the evaluation of the different properties. However, most of the authors reported attractive results such as high strength and hardness [22,23,43,44,45], high-temperature oxidation resistance [46,47,48,49,50], high corrosion resistance [51,52,53,54], high temperature wear resistance [21,27,41,50,54,55,56,57,58,59,60,61,62,63,64,65,66,67], unique electrical and magnetic properties [68,69,70,71] and higher biocompatibility [72,73,74,75]. Owing to these superior combinations of mechanical and thermal properties, high-entropy coatings (HECs) have been proposed as a prominent solution to mitigate high-temperature tribological issues [41,56,64,67,76,77,78,79,80,81]. 

**Figure 3:**
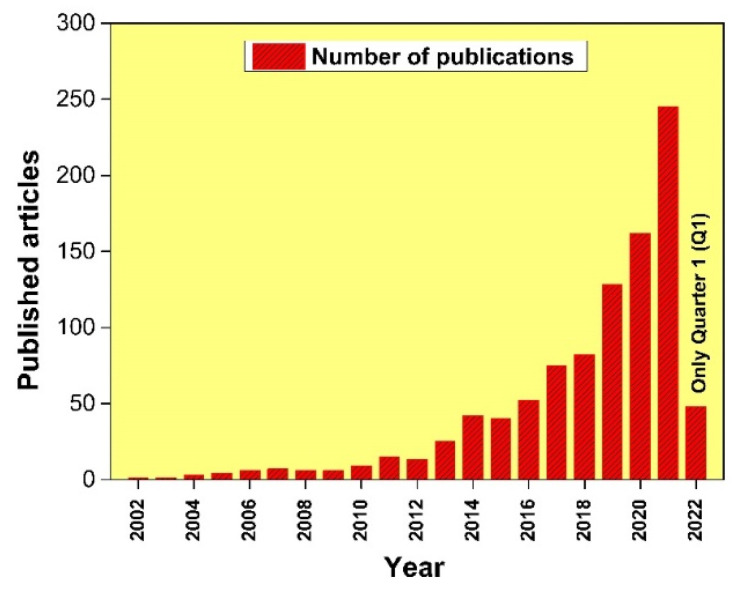
Number of papers published related to high-entropy coatings (HECs) from 2002 to 2022 (exported with Scopus analysis tool).

**Figure 4:**
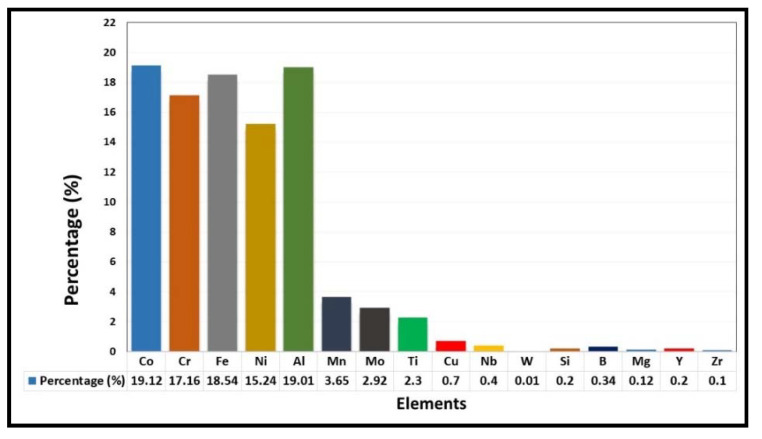
Element selection occurrence in high-entropy alloys used as a feedstock for various coating processes.

Figure 3 demonstrates the number of published articles related to HECs in several years from 2002 to 2022 (considered only first quarter of 2022), exported with Scopus advanced search analysis tool. It can be observed from Figure 3, the major research was started on the high entropy (HE) coatings in the year 2004, and then in the year 2021, the number of publications was raised to more than 250. Several authors [27,41,54,55,56,57,62,64,67] have investigated the tribological behaviour of various HEA systems by varying or replacing one/more element(s) of cantor (CrMnFeCoNi) alloy. Figure 4 demonstrates the elements used as a feedstock in various processes and the majority of HEAs have a base comprising CrFeCoNi with the primary addition of Mn, Al, and Mo and minor addition of some inclusion such as Si, TI, Nb, W, Zr, B and Cu has also been reported to achieve desired properties. In most of the cases, elements have been selected based on the end application such as Cr, Al, Si, Ti for oxidation resistance, for corrosion resistance Cr and Ni, B2-forming Ni-Al/Co/Fe for friction and wear resistance and Ni/Co-based FCCs for plasticity [82]. Their performance has influenced the selection of these alloys for HEA feedstocks in other processing routes, such as casting and other deposition techniques. Some studies directly focused on the tribological properties of the Cantor alloy, while a handful further modify the Cantor alloy by adding ratios of Ti, C, Nb, Al, or Cu to it.

Another common modification of the Cantor alloy for high-entropy alloy development is replacing one base element with another; replacing Mn with Al was a common variation in the publications. The replacement of Mn with Al promotes the transition of FCC to BCC phase, and which induces higher strength. In single-phase HEAs, it is difficult to reach a reasonable balance between strength and tensile ductility [83]. Research has shown that single-phased FCC structured HEAs are ductile [84], whereas, single-phased BCC structured HEAs can be very strong but at the price of brittleness [25]. Such single-solid-solution HEAs could be a potential candidate for a variety of industrial applications in bulk and coatings form at various temperatures range. As discussed previously, the necessity for the industrial applications has triggered the development of such coatings which possess the excellent combination of mechanical and thermal properties and survive under demanding service conditions. The failure of such industrial components is mainly attributed to the surface phenomena such as friction, wear, oxidation and corrosion [85,86,87]. The development of these HE coatings to tribological interfaces can provide the protection against friction and wear, corrosion, high thermal residual stresses, local heating, or oxidation etc., which significantly improve the performance with diminished operating costs. The high-entropy coatings (HECs) are divided into three categories based on the already emerged classification [34,38] and the state-of-the-art for HEMs and their related coating materials:

- **High-entropy metallic coatings**, including the transition metal-based HECs (contain elements like Al, Co, Cr, Cu, Fe, Mn, Ni, Ti, and V) and the refractory HECs (contain elements with high melting temperature, such as Cr, Hf, Mo, Nb, Ta, Ti, V, W, and Zr,) [34]. The refractory HECs are designed to prevent the substrate materials from high-temperature oxidation, abrasion, wear and corrosion [38,88].
- **High-entropy ceramic coatings**, e.g., HEAs mixed with oxygen, boron, and other anions. The components are mainly composed of strong nitride/carbides/oxides-forming elements such as Al, Ti, Cr, Si, Nb, Zr, etc. The incorporated N, C, and O in HECs are not the component because the coatings are the mixture of the constituent binary nitrides/carbides/oxides which are still in the state of solid solution with high-entropy effect. These high-entropy ceramic coatings are reported to possess outstanding surface properties, such as high hardness, thermal stability, corrosion resistance, and low diffusivity, which have great potentials in hard protective coatings and diffusion barriers [38]. Particularly, reactive magnetron sputtered high entropy (HE) nitrides have received global attention as a new type of protective coating with excellent mechanical properties. Since the nitrogen content of the films has a strong influence on the structure and mechanical performance of the HE nitrides, several studies reported the HE nitride films and coatings deposited by re-active sputtering at varying nitrogen flow ratios (RN). Only one broad peak was identified in these XRD patterns when depositing with a N2 flow of 0 SCCM (standard-state cubic centimetre per minute), indicating that the coatings had an amorphous structure. HE nitride coatings with a basic FCC structure could be produced as the N2 flow increased. The effects of non-nitride-forming element(s) on the microstructures and mechanical properties on HE nitride films/coatings are still not clear, which needs further studies and is helpful to understand the strengthening mechanism of the HE nitride films and coatings and develop the new HE nitride systems with higher hardness.
- **High-entropy composite coatings**, in which the HEAs could act as the matrix/binder for hard ceramics and the reinforcements in lightweight alloys, such as Al and Mg alloys. Hard ceramic reinforcements (e.g., TiN, NbC, TiC, and TiB_2_) [89,90,91] with high melting temperature (T_m_) and hardness, excellent wear resistance, and chemical stability, as well as the good metallurgical bonding with HEAs matrix have been synthesized into the high-entropy composite coatings to enhance the surface performances further. To date, some progress has been achieved on high-entropy composite coatings with outstanding properties, such as the TiN/CoCrFeNiTi, NbC/AlCoCrFeNi coatings fabricated by laser cladding and the TiC–TiB2/CoCrCuFeNi coatings synthesized by the in situ plasma transferred arc cladding [38]. Besides hard ceramic reinforcements, the hard Ni60 powders were also adopted as the reinforcements and incorporated into the AlCoCrFeNiTi coating through plasma spraying, by which the hardness and wear resistance at elevated temperature of the sprayed coatings were clearly enhanced [38,66]. Therefore, the surface modification and coatings development with controlled microstructure and properties are the subject of intensive research [92,93,94].

Currently, most authors have developed HECs by using different coating methods such as magnetron sputtering [95,96,97], laser cladding [98,99], induction cladding [100], plasma spraying [54,69,70,97], electrodeposition [101], high velocity oxy-fuel (HVOF) [41,50,51,54,55,56,59,61,65], cold spraying [102,103,104,105] and many others. Moreover, numerous methods are available to prepare the powder before cladding/spraying to get into a uniform size and shape, including atomization, mechanical alloying, and ball milling. Similarly, HE films can be deposited by magnetron sputtering using target materials in three different ways, which is further discussed in Section 5. The majority of the research has been done on thermally sprayed and cladded HECs. However, there is very limited information available related to HE films/coatings developed by the vapor deposition and cold spray methods. Therefore, further investigation is needed to optimize the process parameters and explore more manufacturing processes which can be useful to produce HEA wires and targets [102,103,104]. 

In the years 2016 and 2017, Ye et al. [37] and Yan et al. [106] reviewed and briefly discussed the thermodynamics, and the main four core effects for novel behaviour, manufacturing processes, process parameters, properties and applications of HECs. Recently, in 2018, Wei Li et al. [33] reviewed the HECs very critically and outlined the relationship between microstructure and properties, coating methods, different HEMs systems, the effect of the addition of different elements, and mechanical behaviour of HECs, in the year 2020, Meghwal et al. [82] explored and summarized the different aspects of thermal sprayed HECs in terms of feedstock preparation, different high-entropy coating systems and their properties in the review paper.

**Figure 5:**
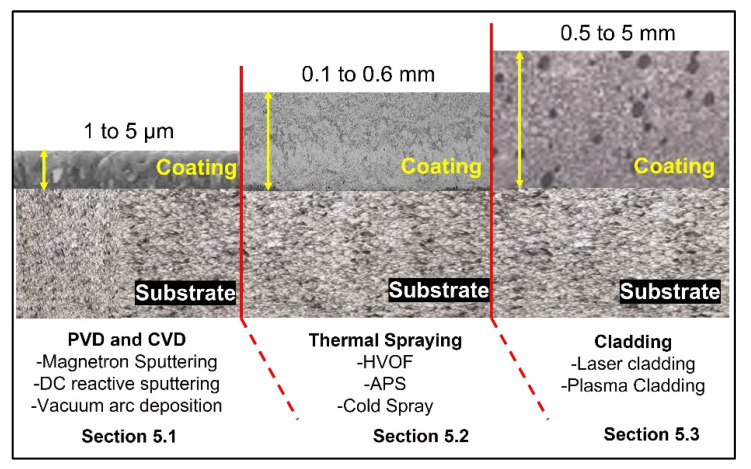
Development of high-entropy coatings (HECs) using various synthesis methods.

Relative to the aforementioned review papers, this review article focuses on the microstructure and tribological properties of HE coatings developed by various synthesis methods (as shown in Figure 5) such as vapor deposition methods, thermal spraying and cladding, etc. The authors also have summarized the fundamental aspects of HEA alloys, the fabrication routes for HEAs and high-entropy coatings (HECs) and tribological properties under various conditions. The tribological behaviour of high-entropy alloys cannot be described in a general way, and each system must be analysed separately depending on the type of wear involved, the HEA system and the operating conditions, and other variables. Adhesion and abrasion have been identified as the most dominant wear mechanisms in high temperature tribological systems, but the detailed understanding of the mechanisms is still inadequate. Considering this variability and lack of understanding, it can be concluded that more extensive and interdisciplinary tribological investigations at high temperatures are necessary. Even though the research on the high temperature tribology of HECs has significantly grown during the last several years, there needs to be a better understanding of this complex and challenging field. Therefore, the focus of this review article is to improve, expand and create new knowledge and understanding of the wear-related phenomena of different HECs developed using various synthesis methods. 

## 3. Fabrication Routes for HEAs

### 3.1. HEAs in the Form of Targets and Fibres

Physical vapor deposition (PVD) and chemical vapor deposition (CVD) are commonly used in the deposition of thin-film coatings by transferring target materials onto the surface of substrates. Recently, PVD and CVD methods such as magnetron sputtering, reactive magnetron-sputtering, and vacuum arc deposition were reported to fabricate high-entropy coatings with desired surface properties, in which high energy-processes (including resistance heating, arc, and ion bombardments) were used to sputter the HEA-based target materials with multiple elements. Li et al. [38] recently reported three distinct methods for depositing HEA coatings using magnetron sputtering, which are addressed further in Section 4.

The most common synthesis methodology for HEAs in the form of rods or ingots is liquid-state processing, often known as the melting and casting method, which can be performed using either of two processes: vacuum arc melting or induction melting. HEAs produced using these approaches accounted for over 75% of the work published so far on high-entropy alloys [107]. So far, the arc melting technique has been the dominant method for producing HEAs (in bulk form) because high temperatures can be achieved by varying the electrical power provided to the electrodes. Temperatures more than 3000 °C are possible, which can melt the majority of the metals commonly employed in the production of HEAs. This process is beneficial for producing HEAs from elements with high melting temperatures, such as refractory elements such as Ti and Zr. The drawback for some metals with low melting temperatures, such as Mg and Zn, is the possibility of evaporation. Resistance and induction heating furnaces are used to produce HEAs from such elements because they have greater compositional control over the arc melting process [21]. After the melting process is completed by either method, the HEAs are immediately cast into various shapes such as rods, wires, and others for diverse applications [108,109].

Another approach for producing bulk HEAs is the solid-state processing method known as mechanical alloying (MA) following spark plasma sintering (SPS). The diffusion of distinct species into one another to produce a homogenous alloy is involved in this process. The mechanical alloying process is split up into two steps, the first of which includes the addition of elemental powders used to produce the HEA in ball milling jars, where they are combined and ground to fine powders using a high energy ball milling technique [107]. The powders are then compressed and sintered simultaneously using the spark plasma sintering (SPS) technique. Sintering is performed by a pulsed electric discharge process that produces localized heating for a relatively short period of time and accelerates elemental diffusion processes. To compress and sinter the elements together, a graphite punch is used to apply a uniaxial high pressure at the same time. Typically, the finished HEAs take the form of rods and other symmetrical geometries [110,111].

Furthermore, the hot drawing technique uses tensile forces to stretch HEA rods through a die to produce HEA fibres/wires. Bulk HEAs are heated and softened before being deformed to form fibres. Li et al. [112,113] produce Al0.3CoCrFeNi HEA rods before drawing them into fibres. The tensile strength and ductility of HEA fibres with a diameter of 1.00 mm were outstanding at 298K (1207 MPa and 7.8 %), increasing to 1600 MPa and 17.5 % at 77K [113]. There is a limited literature and knowledge of the production of HEA fibres and wire, therefore, extensive research needs to be done.

### 3.2. HEAs as a Feedstock

**Figure 6:**
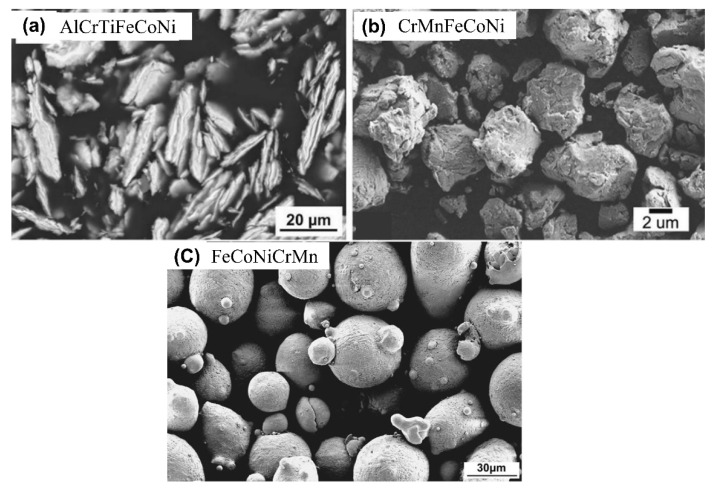
(**a**) Typical powder morphologies observed in mechanically alloyed high-entropy alloys milled in (**a**) argon (dry milling): flaky [114]; and (**b**) toluene (wet milling): irregular [115], (**c**) spherical morphology and satellite particles [104,128].

This article is majorly focused on the high-entropy coatings (HECs) with respect to their high-temperature tribological properties. However, as described in Section 2, several techniques are available to develop the coating on the substrate materials. More specifically, for thermal spraying and cladding processes, preparation of feedstock powder is one of the essential steps to develop the coating with desired properties and maintain a good morphology. However, the morphology of the powder depends on the powder preparation methods. Based on the literature, four synthesis routes have been reported to prepare the HEA feedstocks: blending, arc melting followed by mechanical milling, mechanical alloying, and gas atomization. Figure 6a,b represent the morphology of mechanically alloyed high-entropy alloy powder (AlCrTiFeCoNi) in dry condition [114] and (CrMnFeCoNi) in wet condition [115], respectively. Moreover, Figure 6c demonstrates the morphology of gas atomized FeCrMnCoNi high-entropy alloy powder. Each of the methods has a different impact on the powder phase and characteristics, which could influence the microstructure and properties. Hence, this section mainly focuses on the different powder particle size and morphology produced by various manufacturing methods, systematically described in Table 1. The powder particle size varies with the process to process, which further depends on the process parameters. For instance, mechanical alloying tends to form much finer particles that exhibit nano crystallinity. Whereas, gas atomization provides a wide range of particle sizes, which needs to sieve with the appropriate mesh in a sieve shaker to get the uniform particle size. Table 1 depicts the particle size, morphology and phases present in HEAs powders produced by different methods. Hence, developing a coating with desirable properties is not only depends on the particles size and distribution but also depends on, several process parameters, primarily laser power, scanning speed, and track overlap.

## 4. Fabrication Routes of High-Entropy Coatings (HECs)

A variety of processes are available in the market to produce the HE films/coatings with the desired microstructure and properties. However, the selection of the coating process plays a crucial role in the achievement of excellent properties. There are three main groups for coating processes, including vapor deposition, thermal spraying, and cladding. Each process has a specific effect on the properties of the coatings, such as laser cladding and thermal spraying, which can produce coatings with different morphologies and different thicknesses of the coating. In addition, the distance between the source and the sample, powder feed rate, angle, temperature of the substrate and atmospheric conditions during the spraying of the feedstock powder is equally important for the formation of the dense coating. On the other hand, three different ways to deposited HECs using magnetron sputtering have been reported by Li et al. [38]. First, the high entropy films (HEFs) can be directly prepared by a HEA target, which has a good control over the film stoichiometry and thus, a most often-used way to synthesize the HEFs. Second, HEFs can be fabricated by co-deposition with multiple metal targets and mosaic targets. This technique allows the deposition of HEFs in a wide range of chemical compositions by avoiding the complex target preparation process. Stoichiometry can be controlled by changing the target powers and the relative surface fraction of each element on a given target. Third, HE thin films can be deposited using powder targets. A powder target is easily made by selecting the required metallic powders, weighing, mixing, and finally cold pressing the powder mixture., The authors presented the brief detail of coating methods and their process parameters in Table 2.

Based on the published literature, it is difficult to comment on the suitability of these processes for fabricating HECs. There are several advantages to employing magnetron sputtering, including the ability to combine elements for HEAs directly from single or multicomponent targets. Thermal spray, on the other hand, has the disadvantage of requiring powder and can still result in the typical tortuous microstructure as well as oxidation and phase decomposition challenges. Thus, the coating technique used is influenced by the final microstructure and required properties. One process can produce thick coatings (i.e., CrMnFeCoNi HEA) with acceptable mechanical properties, while the other can produce thin films (high entropy carbides and nitrides) with excellent mechanical properties. Many research papers, however, have been published in which researchers developed HECs using APS and HVOF methods. On the other side, there is relatively little information available on sputtered and cold sprayed coatings, thus further research is required to understand high temperature wear mechanisms. Table 2 provides a general idea of the different coating processes with the characteristics of the coating, which may help in the selection of the coating process. Moreover, cold spraying and plasma cladding processes tend to form thick (1 to 5 mm) and dense coatings, whereas in other processes the thickness range is limited to a few hundred microns. In the following sections, the authors present some of the research finding from a variety of research papers to understand the tribology mechanism of HECs at room temperature to high temperature under different service conditions.

## 5. Tribological Performance of High-Entropy Coatings (HECs) 

The sheer number of industries requiring wear-resistant coatings is staggering. From earth-moving and mining equipment to agricultural machinery such as harvester blades, and from the transmission, steering, and suspension components in automobiles to wear plates in bottling and canning industries, there are ample opportunities for developing high entropy coating solutions for each case-specific scenario. In addition to traditional industries, there is also scope in the renewable energy sector, for example, in wind energy where turbine blades need abrasion resistance from dust and hail, as well as being lightweight yet durable, an ideal case for coatings. Additionally, many industries, especially aerospace and automotive industries, have now adopted HECs, including metallic, ceramic, and composite coatings, for solving problems associated with higher temperatures such as structural stability, high-temperature wear, and oxidation properties. Most of the studies have been carried out by several authors on HECs, mainly investigated and focused on single-phase solid solutions. However, recently, multi-phase microstructures have gained more attraction globally owing to their novel behaviour and properties for tribological applications. There are two ways to improve the tribological performance of HECs, first by forming precipitation of a secondary phase in the parent matrix and another one is by forming an oxide layer during sliding of two surfaces relative in motion, which can act as lubrication at elevated temperature. 

**Figure 7:**
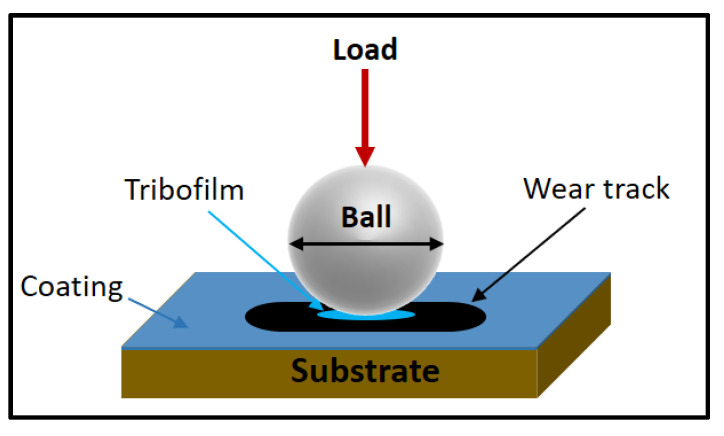
Schematic diagram of tribo-test set up (ball-on-disk) (The black arrow indicates the sliding direction).

A variety of tribo-tests were carried out in the presented papers to measure friction and wear behaviour of HECs. The most used setups are ball-on-disk (Figure 7) and pin-on-disk. In each case, a pin or ball counter face is slid repeatedly either in reciprocating or rotating motion against the developed HE films/coatings. The tribological behaviour was investigated by varying one or more testing parameters such as effects of different speeds, various loads, and with a variation of sliding distance or time. The most common counter face materials used for the tribo-tests were ceramic materials, including silicon nitride (Si_3_N_4_) and alumina (Al_2_O_3_), followed by various steels, including SKH51, 100Cr6, and GCR15. Finally, these tribo-tests produce a wear track on the surface of the coatings. The wear volume was considered in order to calculate the specific wear rate (k) (mm^3^/N.m) as per equation below [154].

(6) $$k=\frac{V}{S.W}=\frac{V}{2.\;f.\;L.\;N.\;W}$$

where V = volume loss (mm^3^), S is the total sliding distance (m), obtained by multiplying the frequency f (s^−1^), stroke length L (mm), and number of cycles (N), and W is the applied load. 

Table 3 incorporates the testing parameters and wear rate of the HECs developed by various methods alongside each system. To compare the results in a better way, the authors normalized some values of sliding distance and speed. Hence, to understand the mechanism of friction and wear from room temperature to elevated temperatures, several articles are reviewed and summarized in this section. It is important noting that some of the wear experiments were conducted using different tools/parameters; these results may not be comparable, but they are useful in understanding the tribological performance of various HECs.

### 5.1. Vapor Deposition and Related Methods

Vapor deposition (VD) methods are widely utilized in the deposition of thin-film coatings by transforming target materials onto the surface of substrates. In this section, the authors have summarized the outcome of various studies investigated the tribological performance of HEFs developed by magnetron sputtering. PVD and CVD methods, such as direct current magnetron sputtering, reactive magnetron sputtering, and vacuum arc deposition, have recently been reported to fabricate HEFs with desired surface properties, in which high energy processes (such as ion bombardments, arc, and resistance heating) were used to sputter the HEA-based target materials with multiple elements.

**Figure 8:**
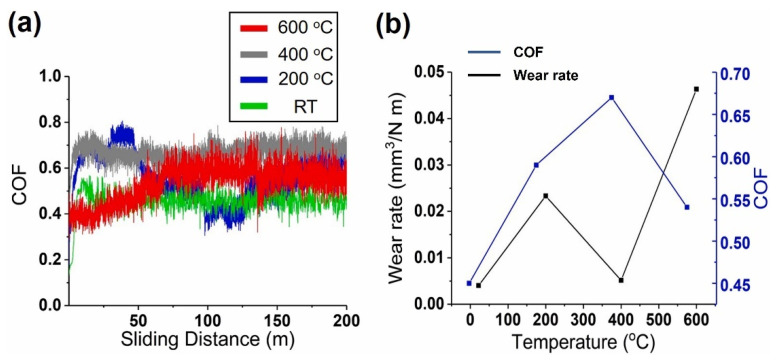
Friction and wear tests at different temperatures with (**a**) plot of COF versus sliding distance, and (**b**) plot of wear rate and COF versus the tribo-test temperatures [155].

S. Alvi et al. [155] fabricated and investigated the hardness and wear properties of CuMoTaWV coating deposited by using spark plasma sintering. The as-sprayed HEA coating comprised a BCC solid solution with a hardness of 600 HV and V-rich zones with Vickers hardness value of 900 HV. The authors investigated the wear performance of high-entropy coating (HEC) from RT to 600 °C in sliding motion against Si_3_N_4_ counter ball. Tribological studies showed adaptive behaviour of the alloy at RT, 400 °C and 600 °C, while at 200 °C, galling wear was observed (Figure 8). At RT, the lowest COF and wear rate was observed due to the formation of the Ta- and W-rich tribo layer in the wear track. As temperature increased from RT to 200 °C, adhesive wear through galling led to an excessive material transfer to the counter ball. There was a reduction in wear rate at 400 °C owing to the oxidation of Cu resulted in the formation of adaptive CuO, along with the unaffected high-hardness V-rich phase. At elevated temperature (600 °C), the formation of CuO and excessive oxidation of V-rich phase resulted in a low COF but slightly higher wear rate. Alvi et al. [156] synthesized the CuMoTaWV film by means of magnetron sputtering methods from a single partially spark-plasma-sintered targe in subsequent studies. The authors evaluated the mechanical and tribological properties of these sputtered films. The CuMoTaWV film showed an average hardness and nanopillar compressive strength of 19 ± 2.3 and 10 ± 0.8 GPa, respectively, which are ∼20% higher than those of the HEFs reported in the literature.

**Figure 9:**
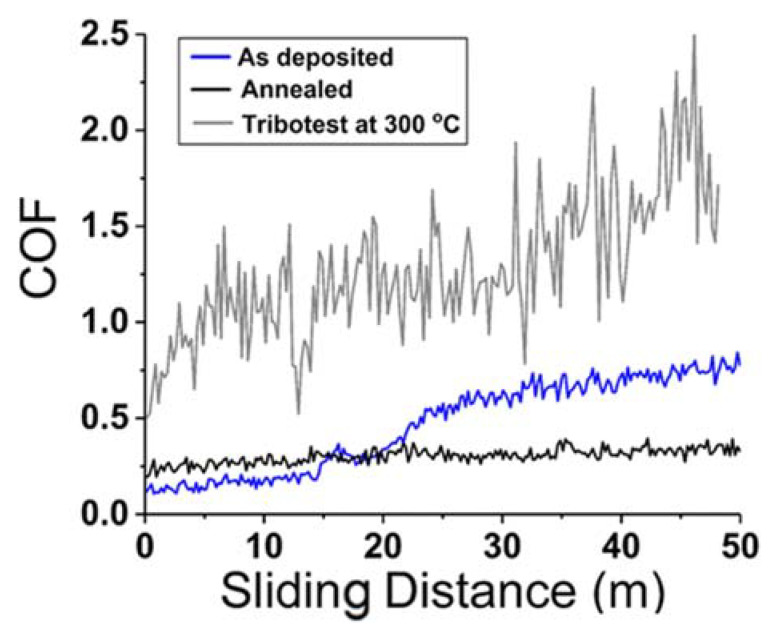
Friction versus sliding distance plot of as-deposited, annealed (300 °C), and 300 °C test temperature CuMoTaWV high-entropy films [156].

The high hardness and compressional strength of the reported film were attributed to nanocrystalline grain size and grain-boundary-controlled plastic deformation. The wear behaviour and adhesion of the CuMoTaWV film on a steel substrate were improved by annealing at 300 °C, and it showed improved coefficient of friction and wear resistance at RT and 300 °C. The reported results suggest that HEFs can be beneficial for wear and nanopillar applications. Figure 9 demonstrates the friction coefficient of as deposited and annealed CuMoTaWV films. The authors concluded that the frictional and wear behaviour of the annealed high-entropy film can be related to its high hardness, nano crystallinity, and better adhesion.

**Figure 10:**
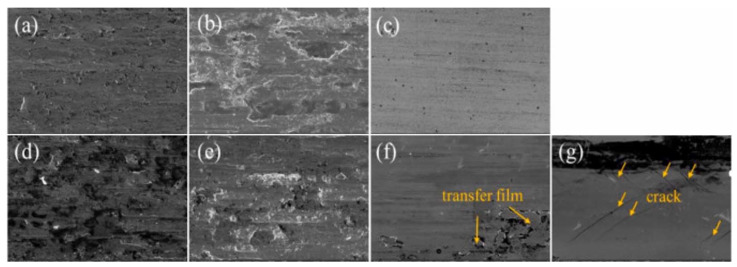
SEM micrographs of wear scars of AlCrTiV, AlCrTiV-N, AlCrTiVSi and AlCrTiVSi-N films against GCr15 and Al_2_O_3_: (**a**) AlCrTiV@GCr15, (**b**) AlCrTiV@Al_2_O_3_, (**c**) AlCrTiV-N@GCr15, (**d**) AlCrTiVSi@GCr15, (**e**) AlCrTiVSi@Al_2_O_3_, (**f**) AlCrTiVSi-N@GCr15, and (**g**) AlCrTiVSi-N@Al_2_O_3_ [157].

Cai et al. [157] investigated the effect of plasma nitriding on the microstructure and tribological behaviour of AlCrTiV and AlCrTiVSi films, prepared by DC magnetron sputtering. After plasma nitriding, the elastic modulus and nano-hardness of AlCrTiV and AlCrTiVSi films are increased, which corresponds to the improvement of their tribological property against GCr15 and Al_2_O_3_. The AlCrTiV film with plasma nitriding has the lowest friction coefficient (against Al_2_O_3_) and the best wear resistance (against GCr15 and Al_2_O_3_), owing to its perfect balance of hardness and toughness. Without plasma nitriding, the wear mechanism of AlCrTiV and AlCrTiVSi HEFs against GCr15 and Al_2_O_3_ is mainly adhesive wear, accompanied by slight abrasive wear and oxidative wear. After plasma nitriding, for AlCrTiV film, the wear mechanism against GCr15 is slight adhesive wear, and that against Al_2_O_3_ is slight wear; while for AlCrTiVSi film, the wear mechanism against GCr15 is slight adhesive wear accompanied with oxidative wear, while that against Al_2_O_3_ is fatigue wear as shown in Figure 10.

Tuten et al. [130] fabricated the multi-component equimolar TiTaHfNbZr HE films on Ti-6Al-4V substrates by RF magnetron sputtering. The sputtered films exhibited a homogenous surface topography with a fine grained amorphous structure, providing a significant enhancement of the mechanical properties. Specifically, significant increase of hardness and elastic modulus of the surface coating led to an enhancement of the tribological properties, such as wear resistance and coefficient of friction, which dictate the suitability of coatings for various biomedical applications. The authors expounded that the equimolar TiTaHfNbZr HEFs can serve as an effective protection against wear and cracking especially for long-term orthopaedic implants.

**Figure 11:**
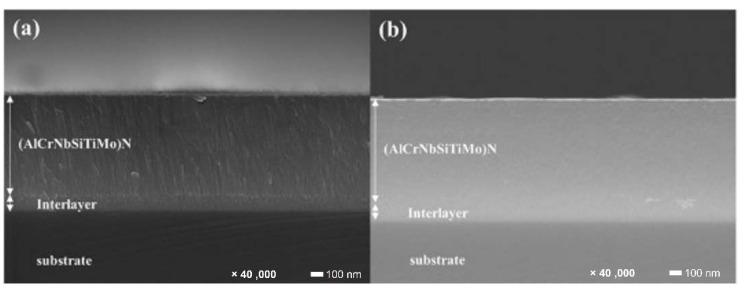
FE-SEM cross-sectional image of AlCrNbSiTiMoN coatings at (**a**) 0 V, (**b**) −200 V [79].

Lo et al. [79] investigated the characteristics of (AlCrNbSiTiMo)N coatings fabricated using RF magnetron sputtering by tuning substrate bias. Figure 11 demonstrates the coating microstructures of (AlCrNbSiTiMo)N coatings developed at bias (a) 0 V and (b) −200 V. All coatings deposited at 300 °C substrate temperature showed favourable mechanical performance, unanimously. By varying substrate bias, the hardness significantly increased from 28.2 GPa to 34.5 GPa. The promotion of mechanical performance was ascribed to the strengthening effects of coatings, including grain boundary strengthening, denser structure, and more ion-induced defects, when substrate bias was applied. The self-lubricating MoO_3_ played an important role in the reduction of friction coefficient at elevated temperature wearing process. As compared to the room temperature wear test, the friction coefficient of coatings showed a remarkable drop at elevated temperature.

On the other hand, the coatings at −100 V performed at the lowest wear rate of 1.2 × 10^−6^ mm^3^/N.m, while it was below 5 × 10^−6^ mm^3^/N.m for the other coatings. The outstanding tribological performance of the coatings was associated with different types of wear mechanisms of wear tracks. The balance between improving the H^3^/E^2^ value and controlling the compressive residual stress by tuning substrate bias was also demonstrated for an optimal anti-wear capability. Similarly, Lai et al. [158] evaluated the microstructural and tribological properties of the (AlCrTaTiZr)N coating influenced by the substrate bias voltage, ranging from 0 to −200 V in the reactive magnetron sputtering process. Reduction in wear rate attributed to the increment of the substrate bias, while the friction coefficient almost kept constant at 0.75. With the bias voltage of −150 V, the authors obtained the lowest wear rate of 3.65 × 10^−6^ mm^3^/N.m for the coating, which could be explained by the high hardness and cohesive strength due to the enhanced ion-bombarding effect.

**Figure 12:**
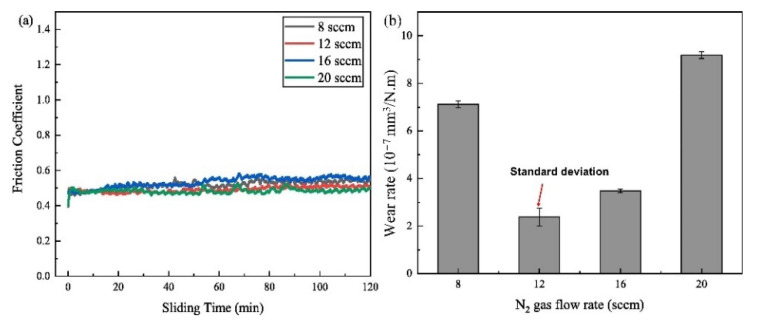
(**a**) Friction coefficients of the (AlCrTiVZr)N films as a function of the sliding time, and the inserted figure shows the average friction coefficient of the (AlCrTiVZr)N films as a function of the FN. (**b**) Wear rates of the (AlCrTiVZr)N films as a function of the FN [160].

Si et al. [159] prepared the TiVCrZrWN_x_ coatings by magnetron sputtering at different nitrogen flow (FN) rates. With the increase of nitrogen flow rate, the crystal structure of TiVCrZrWNx coating changes from an amorphous state to an FCC. The authors also investigated the corrosion and friction behaviour of these sputtered coatings. In the friction resistance test, the self-lubricating V_2_O_5_ phase is formed in the rolling friction process, and its friction coefficient is greatly reduced to 0.38. Moreover, the authors expounded that the TiVCrZrWN_x_ coatings could possess excellent mechanical properties, corrosion resistance, and friction resistance, which is an indispensable technology in industrial production technology. It provides a novel idea for designing a unique protective coating for advanced marine equipment and other industrial fields. Similarly, Xu et al. [160] also fabricated the (AlCrTiVZr)N films using high power impulse magnetron sputtering (HiPIMS) at various nitrogen flow without additional heating. According to the XRD observations, the (AlCrTiVZr)N films present a simple FCC solid-solution phase. At FN = 12 sccm, the films have super-hardness of 41.8 GPa and low wear rate of 2.3 × 10^−7^ mm^3^/N.m as shown in Figure 12. 

Wang et al. [161] deposited (CrNbSiTiZr)C films using an equimolar CrNbSiTiZr alloy target by reactive RF magnetron sputtering and investigated the tribological behaviour at room temperature. The (CrNbSiTiZr)C film has an NaCl-type structure with nano-size crystalline grains with an approximate size of 5 nm. The authors reported that the hardness and Young’s modulus of the (CrNbSiTiZr)C film reached 32.9 GPa and 218.7 GPa, respectively. Moreover, the (CrNbSiTiZr)C film has a minimum friction coefficient of 0.3, the best wear rate of 4.2 × 10^−6^ mm^3^/N.m due to the formation of a transfer film, as well as the high H/E (0.151) and H^3^/E^2^ (0.747) ratio. 

### 5.2. Thermal Spraying

Based on the research findings [65,69,138], thermal spraying is more advantageous and feasible for producing coatings with lower degree of dilution and desired properties. The HECs developed using thermal spray methods, have shown good microstructural stability with excellent mechanical properties. Thus, thermally sprayed HECs could be promising candidates for high temperature tribological applications. This section mainly puts an emphasis on the tribological properties of thermally sprayed, including atmospheric plasma spray, high velocity oxy-fuel and cold spray, HECs, some of which are reviewed in Table 3. 

**Figure 13:**
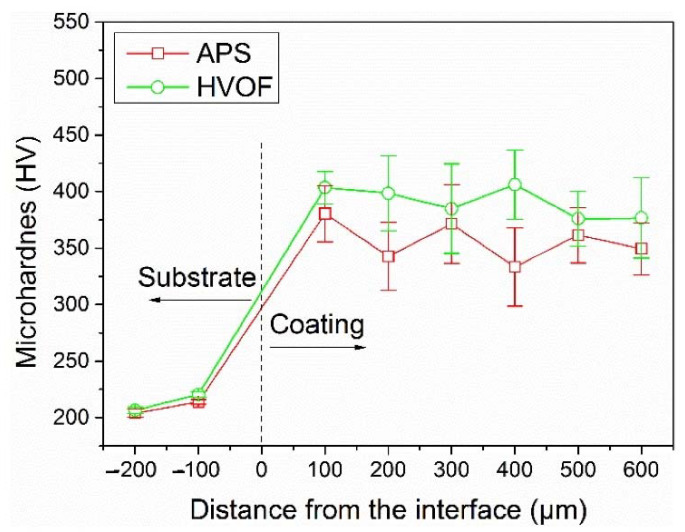
Microhardness profiles along the cross-section of the HECs [41].

**Figure 14:**
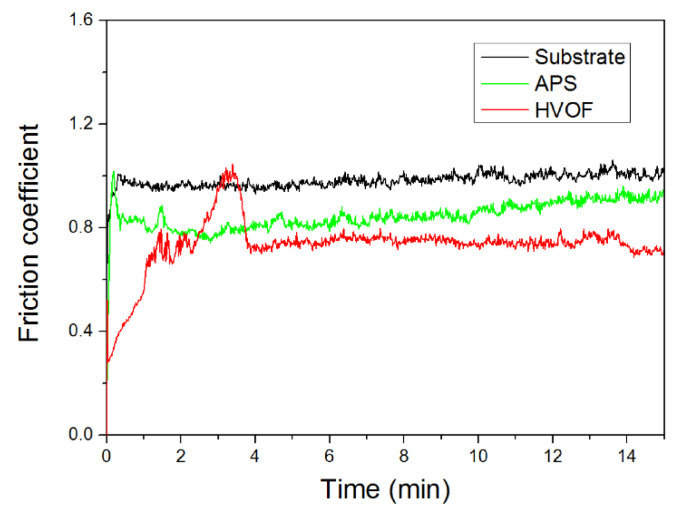
The friction coefficients of the APS coating, the HVOF coating, and the 45# steel substrate [41].

Li et al. [41] evaluated the wear performance of the FeCoCrNiMo_0.2_ coating fabricated with APS and HVOF spray techniques. Both the APS and HVOF coating were mainly composed of an FCC solid solution phase with a small number of oxides, which were identified as a mixture of Fe_3_O_4_, Fe_2_O_3_, and some binary oxides of the chemical formula AB_2_O_4_ (A = Fe, Co, Ni, and B = Fe, Cr) in a spinel structure. The average microhardness of the APS coating and HVOF coating was 356.4 HV_0.2_ and 390.9 HV_0.2_, respectively. The results indicated that these oxides also have a large solid solubility in each other with the same phase structure due to the high entropy effect. The volume wear rate of the APS coating was about 3.9 × 10^−5^ mm^3^/N.m, which was significantly lower than the HVOF coating (4.8 × 10^−4^ mm^3^/N.m) and the substrate (5.4 × 10^−4^ mm^3^/N.m). Figure 13 and Figure 14 show the microhardness graph and friction coefficient of a HECs developed using the APS and HVOF methods, respectively. The breaking of the oxide film that developed on the worn surface of the APS coating was mostly responsible for the volume loss of the APS coating during the wear test, whereas mild abrasive wear mechanism was dominated for the HVOF coating.

**Figure 15:**
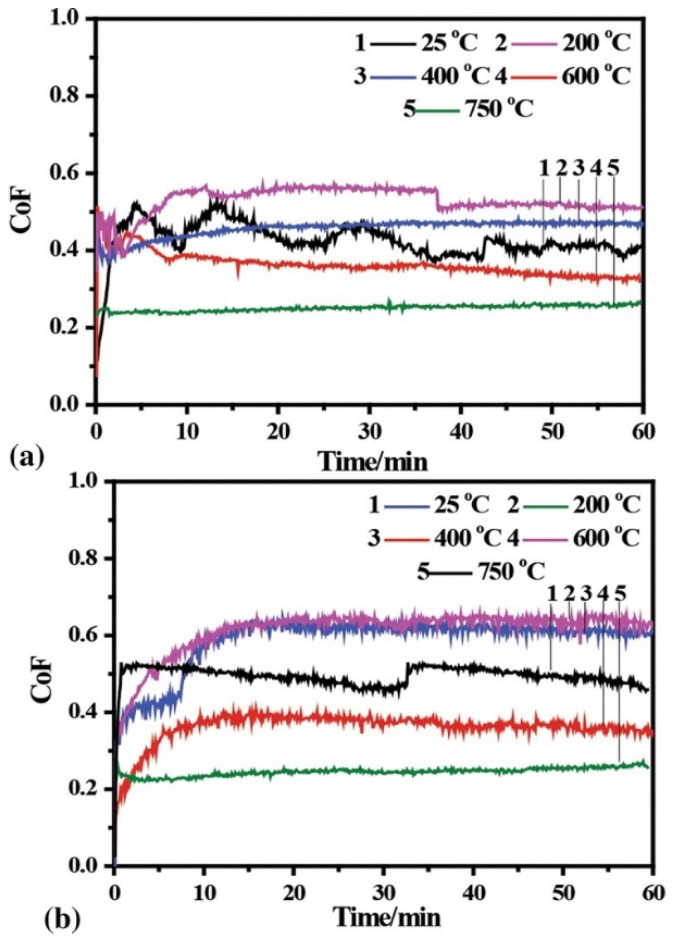
Friction coefficient curves of (**a**) Al_0.2_Co_1.5_CrFeNi_1.5_Ti + Ag coating and (**b**) Al_0.2_Co_1.5_CrFeNi_1.5_Ti coating at different temperatures [77].

**Figure 16:**
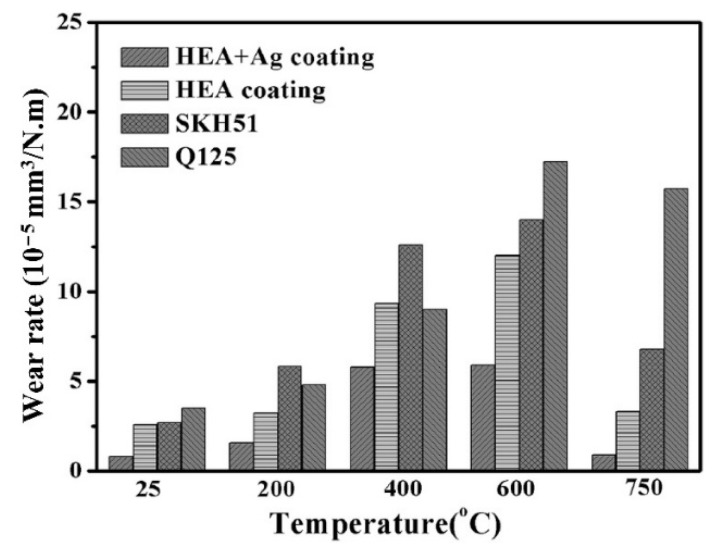
Wear rate of composite coatings and SKH51, Q125 at different temperatures [77].

Li et al. [77] studied the microstructure and tribological properties of plasma-sprayed Al_0.2_Co_1.5_CrFeNi_1.5_Ti-Ag coating formed on carbon steel from 25 to 750 °C. Additionally, they investigated the effect of the addition of Ag in HEC. The as-sprayed HEC and HEA-Ag coating comprised the major FCC solid solution with minor BCC. At low temperatures (RT to 400 °C), the tribological properties of HEA-Ag composite coating depend on their mechanical properties and the lubrication of Ag. At high temperatures (600–750 °C), both the HEA-Ag and HEC obtained good tribological properties; in particular, a relatively low friction coefficient (0.253) and wear rate (0.89 × 10^−5^ mm^3^/N.m) was achieved at 750 °C for HEA-Ag coating, systematically represented in Figure 15 and Figure 16, respectively. The authors reported that the lubricious film that was formed consisted of Ag and various oxides on the frictional surface, which contributed to the outstanding tribological properties. Hence, the addition of Ag had little effect on the friction coefficient of coating at high temperatures. Finally, a lubricious film that was formed consisted of Ag and various oxides on the frictional surface, which contributed to the outstanding tribological performance. The wear rate of both HEA and HEA + Ag was significantly lower than that of tool steels SKH 51 and Q 125 at all the test temperatures, which indicates the improved tribological properties of HECs.

**Figure 17:**
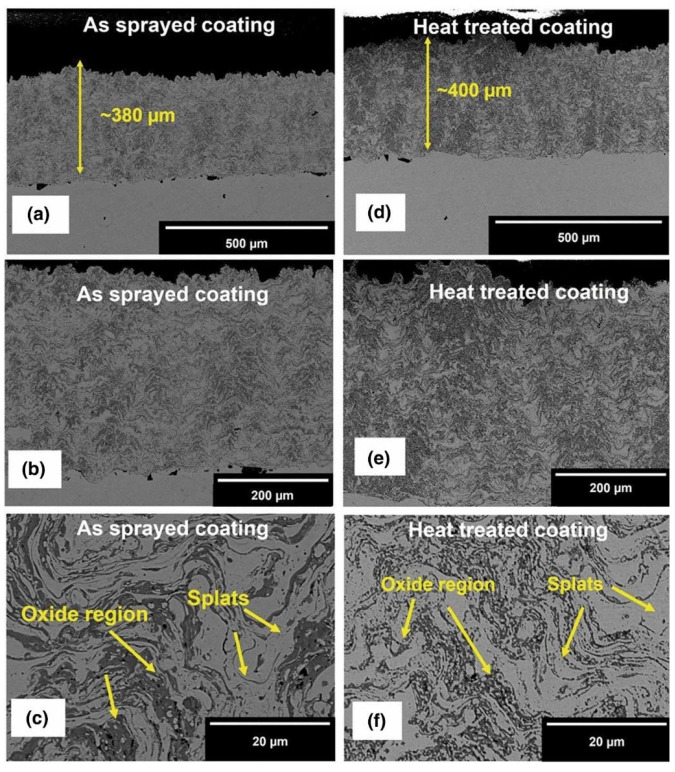
Cross-sectional microstructural images of as sprayed (**a**–**c**) and heat-treated (**d**–**f**) coatings [81].

Xiao et al. [67] investigated the wear behaviour of FeCoNiCrMn coatings developed by plasma spraying at a different H_2_ flow rate. The as-sprayed coating exhibited the FCC solid solution with some oxides content. The authors found that high-temperature annealing plays the role of sintering and promotes the generation of a full metallurgical bond among splats. The annealed coating showed a lower friction coefficient and low wear rate relative to as-sprayed coatings. The improvement of wear resistance by increasing H_2_ flow rate and annealing is attributed to the enhancement of cohesive strength among splats and the increase in oxides. In contrast, Patel et al. [81] developed CrMnFeCoNi coatings by means of HVOF with subsequent annealing heat treatment. The authors concluded that both as sprayed (AS) and annealed (HT) HE coatings exhibited major FCC single solid solution phase and minor formation of oxides as shown Figure 17. 

Both the AS and HT coatings were evaluated against the alumina counter ball on the polished and rough surfaces. The coefficient of friction and wear rate of AS polished and HT polished coatings are almost equivalent, which indicates that there was no significant influence of annealing on the wear performance of the CrMnFeCoNi coatings. However, a just negligible effect can be observed in the friction coefficient results, which were almost the same after some 2000 cycles. The rough surfaces of AS and HT coatings showed high wear rate relative to polished surfaces due to higher initial maximum contact stress and formation higher amount of debris particles with potentially large size. The dominant wear mechanism was adhesive for polished surfaces whereas third body abrasion wear mechanism played vital role for rough surfaces. One of the subsequential studies, Xiao et al. [142] investigated the tribological performance of Al_x_SiCrFeCoNi (where x is 0.5, 1.0, and 1.5 as molar ratios) under both dry and water sliding conditions to simulate the operating environment of compressed air and water pipelines. The volume wear rate of as-sprayed coatings had a decreasing trend with an increase in aluminium content from 0.5 to 1.5 under dry sliding conditions. The wear rate of all the AlxSiCrFeCoNi (where x is 0.5, 1.0, and 1.5 as molar ratios) coatings obtained was slightly higher than previously attained by Tian et al. [121]. The wear rate decreases significantly to 6.7 × 10^−6^ mm^3^/N.m for the Al_1.0_SiCrFeCoNi coating after heat treatment due to the formation of BCC and Cr_3_Ni_5_Si_2_ phases. The formation of grooves, micro pits due to spalling of splats, and micro-cracks at the interface of splats, which all suggest abrasive wear as the primary wear mechanism. In contrast, the wear rate of underwater sliding conditions was lower than under dry sliding conditions due to lubricating as well as cooling effects promoted by the water media. 

Lobel et al. [55] investigated the wear behaviour of AlCoCrFeNiTi coating fabricated on S235 steel samples by the HVOF technique and compared the wear properties with hard chrome. Microstructural investigations of the coating revealed a homogeneous and lamellar multiphase structure with BCC and B2 solid solution. The wear behaviour of the coatings was investigated under various wear conditions in ball-on-disk, oscillating wear, and scratch tests. In comparison with a hard chrome-plated sample, a higher wear resistance could be achieved by applying HECs. Furthermore, no brittle failure occurred under abrasive load in scratch tests, making HEA a potential candidate for wear-resistant coatings. Chen et al. [56] prepared the Al_0.6_TiCrFeCoNi coating on ASTM A572 steel by using HVOF thermal spray technique and evaluated the wear performance of HECs in the range of 300 to 500 °C. The as-sprayed Al_0.6_TiCrFeCoNi HVOF coating possessed a dense microstructure and exhibited two BCC phases with similar lattice parameters, a high microhardness of 789 ± 54 HV_0.1_. The wear behaviour of the coating changed significantly with increasing test temperature. A compacted oxide layer formed on the wear track, which led to the lowest coefficient of friction at T = 500 °C due to its function as a solid lubricant. The authors also reported that after annealing of the HECs at 800 °C, the formation of laves phase ϭ-FeCr in the BCC matrix, which could provide better wear resistance at a higher temperature. 

**Figure 18:**
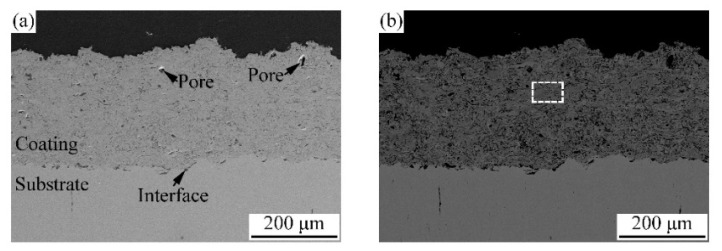

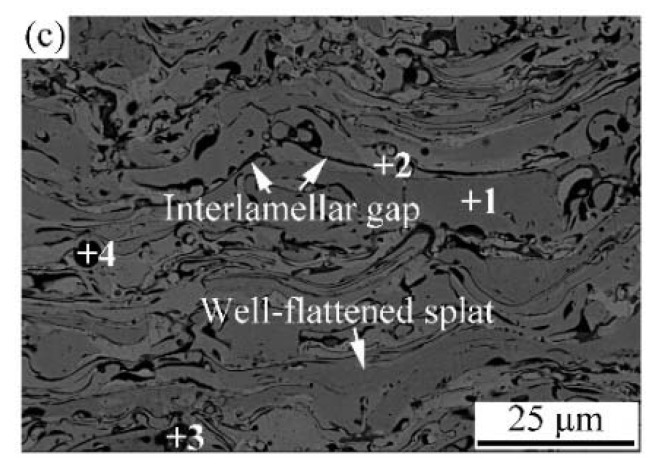
Cross-sectional microstructure of the AlCoCrFeNiSi coating at magnifications of 150× in a secondary electron mode (**a**) and a back-scattered electron mode (**b**) and 1000× (**c**) [121].

Tian et al. [121] fabricated APS AlSiCrFeCoNi coatings (Figure 18) and analysed the wear performance of coating against Si3N4 abrasion media. The coating exhibited a wear rate of 0.38 ± 0.08 × 10^−4^ mm^3^/N.m, which was better in comparison with previously analysed APS AlTiCrFeCoNi coating (0.77 ± 0.01 × 10^−4^ mm^3^/N.m) [64]. Further analysis of wear tracks revealed that adhesive wear and tribo-oxidation wear, as well as slight abrasion wear, were active mechanisms. The superior wear performance of the AlSiCrFeCoNi coating may be attributed to the dense microstructure and the formation of hard tribo films during the wear test. Hsu et al. [117] studied the wear resistance of APS and HVOF AlSi_0.2_Ti_0.2_CrFe_0.2_Co_0.6_Ni_0.2_ coatings. Pin-on-disk wear tests were performed at a load of 1 kg for 20 m. Wear resistance values obtained were 21 ± 3 and 20 ± 2 m/mm^3^ for APS and HVOF coatings, respectively, which were better than conventional bearing steel SUJ2 (12 m/mm^3^). Precipitation of a hard and stable Cr_3_Si phase within the coating contributed to this enhanced wear resistance. Hsu et al. [57] also tested the wear resistance of APS AlSiTi_0.2_Cr_1.5_Fe_0.2_Co_0.6_Ni under similar test conditions [117]. The as-sprayed coating exhibited wear resistance of around 8.5 mm/mm^3^, which increased to approximately 18 mm/mm^3^ after annealing at 800 °C. As in the previous study, precipitation of Cr_3_Si post-heat treatment was responsible for the increase in wear resistance.

**Figure 19:**
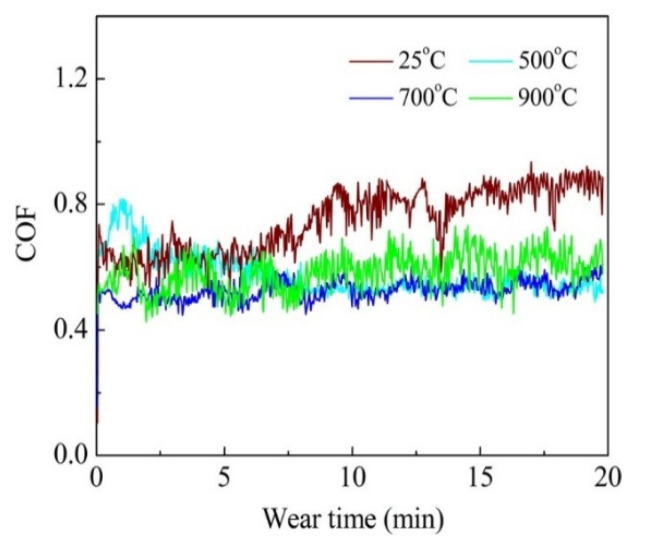
COF of the AlCoCrFeNiTi coating with the change of the wear time at 25 °C, 500 °C, 700 °C and 900 °C [64].

**Figure 20:**
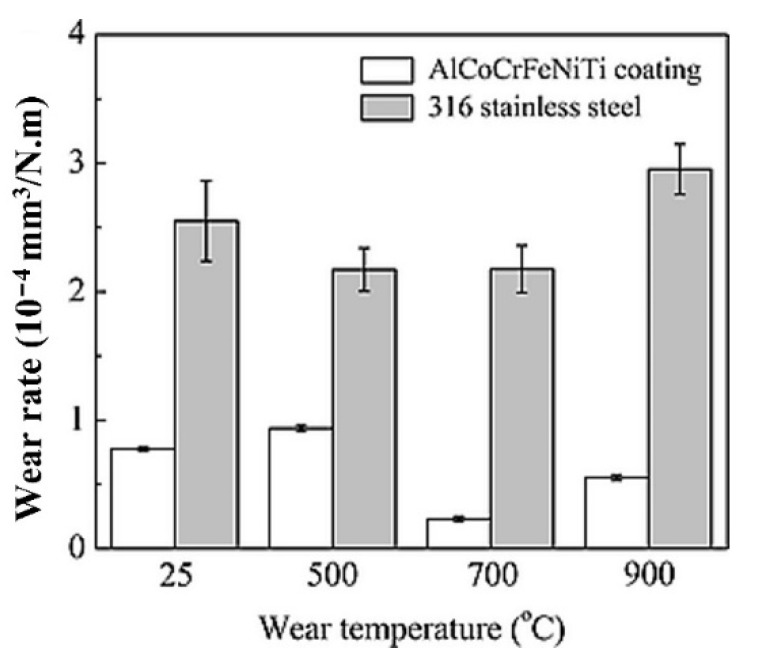
Volume wear rate of the AlCoCrFeNiTi coating and 316 stainless steel at 25 °C, 500 °C, 700 °C and 900 °C [64].

Tian et al. [64] fabricated the AlCoCrFeNiTi coating on the 316 SS substrate by using the APS technique and compared it with the conventional flame sprayed NiCrBSi coating. As-sprayed coating composed of the BCC matrix, minor FCC, and ordered BCC solid-solution phases. The maximum microhardness of the as-sprayed coating was 710 HV, which was about five times that of 316 stainless steels (173HV). The authors evaluated the friction and wear performance of AlCoCrFeNiTi coatings from room temperature to 900 °C, as systematically presented in Figure 19 and Figure 20, respectively. As the temperature increased to 500 °C, the coating suffered more severe adhesive wear, and the wear rate increased. At 700 °C, the coating exhibited the most excellent wear resistance, and the wear rate was 0.23 ± 0.02 × 10^−4^ mm^3^/N.m (Figure 20).

The authors reported the mechanism for lower coefficient of friction at 700 °C and 900 °C; more severe oxidation occurred, and the whole wear surface was covered by uniform tribo films, which can act as a lubricant during the wear testing. Moreover, some oxide and ϭ phases were detected on the wear surface after tests and the coatings showed tribo-oxidation wear and abrasion wear. However, the wear resistance of AlCoCrFeNiTi coating was still better than that of the conventional NiCrBSi coating at the same temperature.

**Figure 21:**
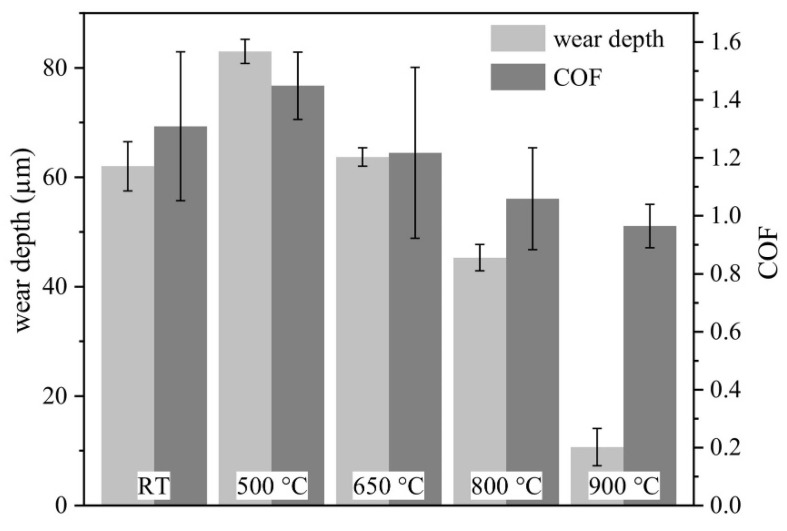
Wear depth and average COF for the high-temperature wear investigations of AlCoCrFeNiTi_0.5_ HVOF coatings tested under reciprocating wear conditions at temperatures of 22 °C (RT); 500 °C; 650 °C; 800 °C and 900 °C [128].

**Figure 22:**
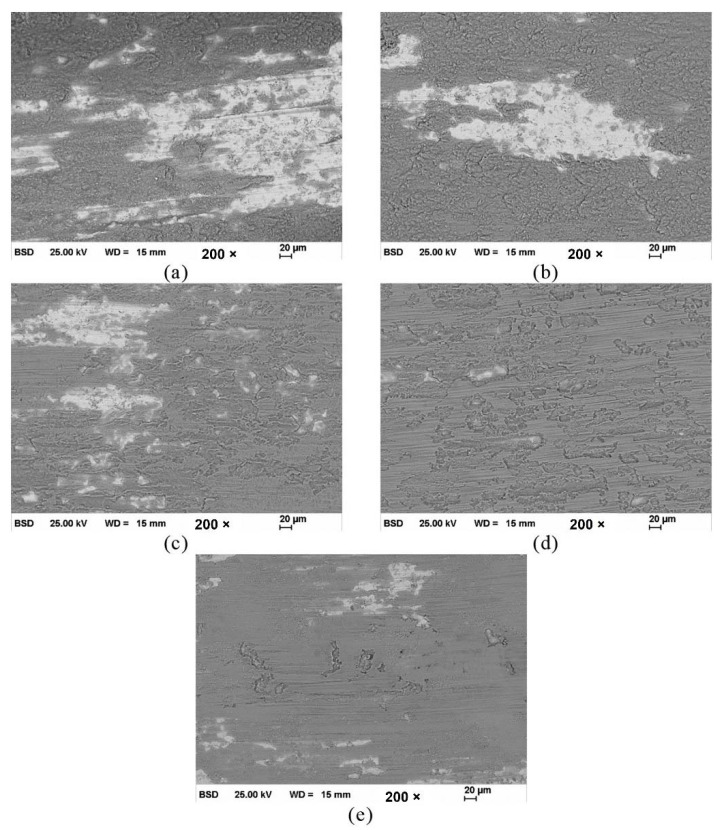
Surface of the AlCoCrFeNiTi0.5 coatings after reciprocating wear tests at a temperature of: (**a**) 22 °C; (**b**) 500 °C; (**c**) 650 °C; (**d**) 800 °C and (**e**) 900 °C [128].

AlCoCrFeNiTi_0.5_ HEA coatings were successfully developed and the wear behaviour investigated at high temperature (>800 °C) by Lobel et al. [128]. Phase analysis also revealed the formation of two major bcc phases. The wear behaviour was investigated in a wide temperature range under reciprocating conditions (shown in Figure 21). After an initial reduction of wear resistance with the increasing temperature, a significant improvement of wear resistance was achieved for temperatures ≥800 °C. Furthermore, a slight reduction in the COF occurs with increased temperature. In comparison with powder metallurgically produced material of the same alloy, improved wear resistance was observed in the whole temperature range. For temperatures ≤500 °C, abrasive wear of the metallic coatings, as well as the formation of loose oxides, was observed. With increased temperature, the increased content of the surface is covered with oxides. For high temperatures ≥800 °C (Figure 22), a compact oxide layer is formed. Grooves indicate abrasive wear behaviour and the protection of the underlying material, resulting in high wear resistance. Phase analysis reveals the formation of additional phases for the highest test temperature (900 °C). The investigations prove the suitability of AlCoCrFeNiTi_0.5_ coatings for high temperature applications. Yin et al. [104] reported on the fabrication and tribological properties of cold-sprayed CrMnFeCoNi. The volume wear rate of the CS coating was 4.76 ± 0.22 × 10^−4^ mm^3^/N.m, with abrasive wear operating as the dominant mechanism. This value was considerably higher than those for APS AlTiCrFeCoNi [56], HVOF Al_0.6_TiCrFeCoNi [121], and APS AlSiCrFeCoNi coatings [66]. 

### 5.3. Cladding

Cladding, including laser and plasma cladding, is commonly used in the production of HECs due to the many advantages of high energy density and strong metallurgical bonding between coating and substrate. Laser cladding has developed into the main fabrication method for HECs, in which pre-placed powders/wires and the thin substrate surface layer are melted and solidified fast under the heat source of a laser. As previously stated, laser cladding typically uses mixed powders as feedstock, which melt under the laser beam and quickly resolidify into a distinctive dendritic structure. With a small degree of coating dilution, sound metallurgical bonding between substrate and cladded coatings can be achieved. In comparison to laser cladding and laser surface alloying, plasma cladding has a larger heat input and higher blowing force, resulting in abundant melting and mixing of the molten coating materials to achieve homogeneity in microstructures and performances. Furthermore, cladded HECs have equiaxed or columnar dendritic morphologies and it is almost defect free. Argon shielding during laser scanning is a standard practice with laser cladding to prevent melt pool oxidation. The tribological properties of HECs synthesized by laser cladding and plasma cladding are explored and summarized in this section.

Jin et al. [162] evaluated the wear performance of the FeNiCoAlCu coating on AISI 1045 steel by using a laser cladding technique. As-sprayed coating composed of FCC and BCC solid solution phases with a typical uniform dendrite microstructure, and the inter-dendritic region and dendritic regions are Fe-rich BCC and Cu-rich FCC solid solution, respectively. The friction coefficient of the coating is in the range of 0.8~0.9 at 200 °C and 400 °C, while the friction coefficient at 600 °C and 800 °C decreased to 0.3, which may be due to the formation of oxide films on the surface of the coatings. The formation of oxide film may be the main reason for the desired high-temperature wear performance. The XPS results indicate that the surface oxidation film of laser-cladded FeNiCoAlCu coating at 800 °C mainly contains Al_2_O_3_, Fe_2_O_3_, Fe_3_O_4_ and CuO. Huang et al. [163] fabricated the TiVCrAlSi coating on the Ti-6Al-4V alloy surface by laser cladding and which was composed of titanium silicide (Ti, V)_5_Si_3_ and BCC phases. The microhardness of the laser cladded coating was found to be close to 1000 HV_0.2_ owing to the formation of precipitation and hard BCC matrix, which significantly improved the wear resistance of Ti-6Al-4V during the dry sliding wear tests [59]. 

**Figure 23:**
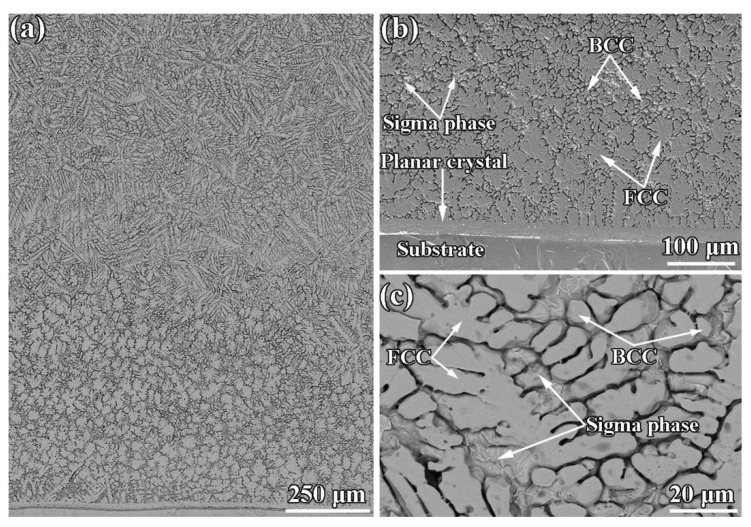
SEM sectional morphology of the (CoCrFeMnNi)85Ti15 coating (**a**) composed of equiaxed columnar dendrites in the bottom-middle region and snowflake-like dendrites in middle-top region; (**b**) planar crystals with the thickness of 20–30 μm is formed at the bottom of the HE coating; (**c**) phases present in the coating microstructure [76].

**Figure 24:**
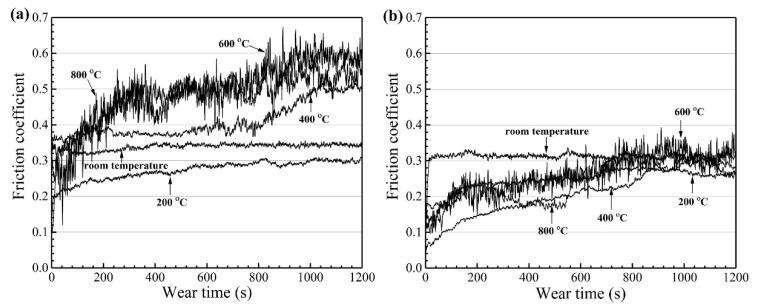
Friction coefficient of the HECs: (**a**) CoCrFeMnNi coating and (**b**) (CoCrFeMnNi)_85_Ti_15_ coating [76].

Wang et al. [76] studied the high-temperature friction and wear performance of CoCrFeMnNi and (CoCrFeMnNi)_85_Ti_15_ coatings formed on Q235 steel by plasma cladding. The CoCrFeMnNi coating is composed solely of an FCC solid solution, while the (CoCrFeMnNi)_85_Ti_15_ coating consists of FCC and BCC solid solutions as well as an intermetallic sigma phase as shown in Figure 23. The (CoCrFeMnNi)_85_Ti_15_ coating shows the best high-temperature wear resistance at 400 °C. The wear resistance of both HECs increases with wear temperatures at temperatures below 400 °C and decreases with wear temperatures above 400 °C. Figure 24 demonstrates the friction coefficient of CoCrFeMnNi and (CoCrFeMnNi)_85_Ti_15_ coatings at different temperatures.

Moreover, the tribological properties of the (CoCrFeMnNi)_85_Ti_15_ coating are nearly 5.5 times those of the CoCrFeMnNi coating during the high-temperature friction process. The wear mechanisms of the CoCrFeMnNi coating are abrasive wear at room temperature, abrasive wear, and slight oxidation wear at 200 °C, and primarily oxidation wear and contact fatigue above 200 °C.

**Figure 25:**
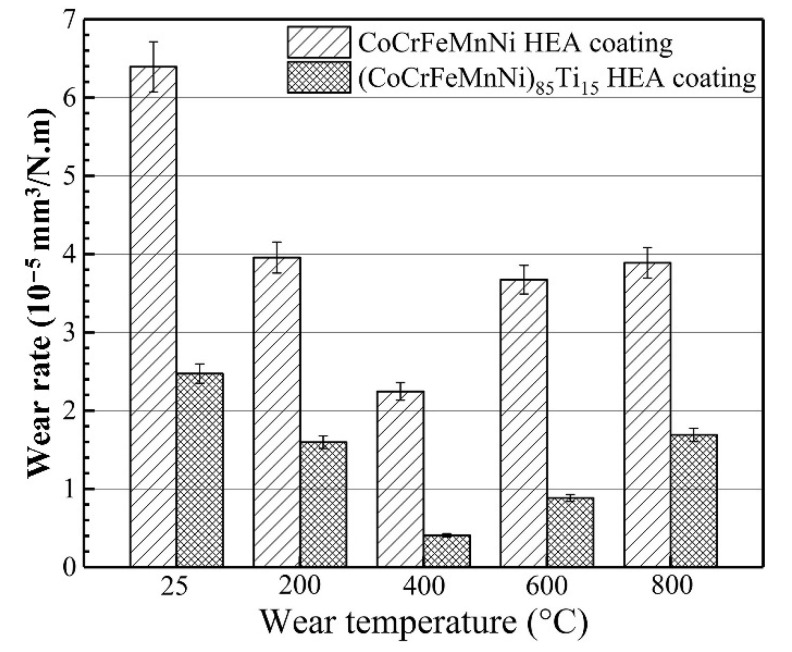
Wear rates of the HECs at different wear temperatures [76].

**Figure 26:**
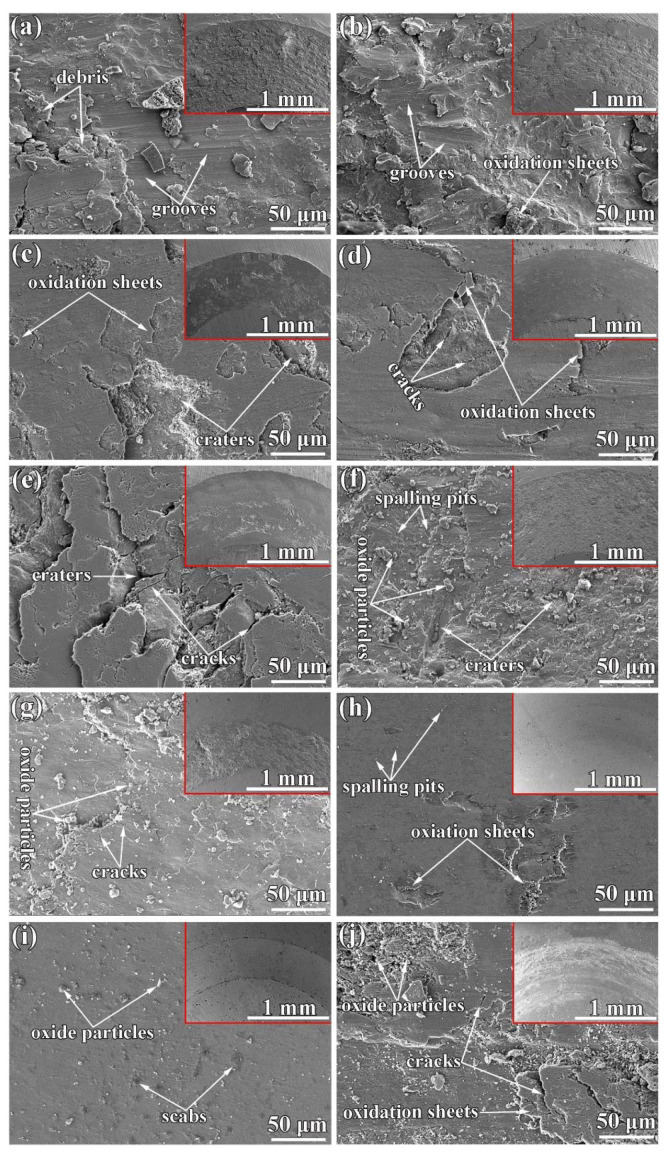
Wear morphology of the HECs: CoCrFeMnNi coating: (**a**) 25 °C, (**b**) 200 °C, (**c**) 400 °C, (**d**) 600 °C, (**e**) 800 °C; (CoCrFeMnNi)85Ti15 coating: (**f**) 25 °C, (**g**) 200 °C, (**h**) 400 °C, (**i**) 600 °C, (**j**) 800 °C [76].

Figure 25 reveals the volume wear rate at a different temperature, where at 800 °C, wear volume rate was 1.8 × 10^–5^ mm^3^/N.m, which was quite higher as compared to at 400 and 600 °C. The (CoCrFeMnNi)_85_Ti_15_ coating exhibits oxidation wear and adhesive wear during high-temperature friction at 800 °C, but at other temperatures, the main wear mechanisms were oxidation wear and contact fatigue, as shown in Figure 26.

Zhang et al. [164] investigated the wear behaviour of the FeCoCrAlCu coating formed on the Q235 steel with about 800 μm in thickness by laser-surface alloying. The microhardness of the coating was around 802 HV throughout the coating. The authors mentioned the three possibilities for higher hardness value, which were solid solution strengthening, rapid solidification and grain boundary strengthening. The mean coefficient of friction of FeCoCrAlCu coating was ~0.58, which was about 66% that of the substrate material. Similarly, both the wear volume and specific wear rate of the coating were an order of magnitude lower than that of the Q235 substrate under a dry-sliding condition.

Cai et al. [165] studied microstructural and wear behaviour of the NiCrCoTiV coatings developed using a combination of laser cladding and laser remelting processes. The results demonstrate that remelted HEC exhibits better wear performance due to high hardness compared to laser cladded coatings. Ti-rich phases and BCC solid solution phase improved the wear resistance of the combination of laser cladding and laser remelting of HECs [165,166,167]. K Huang et al. [168] synthesized Al_0.5_CoCrCuFeNi coating on the magnesium alloy AZ91D by laser cladding and performed the dry sliding wear test at room temperature. The authors found the dry sliding wear resistance of the Al_0.5_CoCrCuFeNi high-entropy coating (HEC) prepared by laser cladding was better than that of the AZ91D matrix, and the wear mechanisms of the two materials were different. The coating showed prominent abrasive wear characteristics, whereas the AZ91D matrix showed obvious adhesive wear characteristics. Jiang et al. [169] fabricated and investigated the wear performance of the CoFeNi_2_V_0.5_Nb_0.75_ and CoFeNi_2_V_0.5_Nb coatings by laser cladding on the 304 stainless steel substrates. The wear resistance of the coatings had been greatly improved as compared to the 304-steel substrate, owing to the combination of the hard Fe_2_Nb-type laves phase and ductile FCC solid-solution matrix. The Fe_2_Nb type Laves phase played a dominant role in resisting the abrasive wear, while the FCC solid-solution phase helped avoid brittle fracture.

Huo et al. [170] developed the CoCrFeMnNbNi coating on the AISI 304 steel by tungsten-inert-gas cladding, which comprised of the FCC solid-solution and Nb-rich Laves phase. The coating provided excellent wear resistance under the condition of dry-sliding wear, due to the hard Laves phase resisted destructive action during sliding and protected the surface against severe elastic deformation. The tough FCC phase protected the surface against the brittle fracture by providing elongation. Y Li et al. [171] evaluated the effect of Cu on the wear behaviour of Al_0.8_CrFeCoNiCu_x_ coating developed on the 5083 Al alloy using laser cladding technique. The authors reported that the addition of Cu decreases the hardness of Al_0.8_CrFeCoNiCu_x_ coatings. However, compared to aluminium alloy, Al_0.8_CrFeCoNiCu_x_ coating still exhibits higher hardness, mainly due to the formation of many BCC phases and lattice distortion effects. The change in Cu content affects the wear resistance and hardness of the Al_0.8_CrFeCoNiCu_x_ coating. With increasing Cu content, the wear rate of HECs also increases, where the mechanisms of Cu_0.5_, Cu_0.75_, and Cu_1.0_ coatings are adhesive and abrasive wear.

Cui et al. [172] developed the FeCoCrNiMnAl_x_ coatings on 4Cr5MoSiV steel by laser cladding and investigated the effects of Al addition on the microstructure and tribological properties. Al element promoted phase transition in FeMnCrNiCoAl_x_ cladding layers from FCC to FCC + BCC, and the BCC phase possessed a typical B2 structure. Al element enhanced the high-temperature wear performance of the FeMnCrNiCoAl_x_ cladding layers and effectively improved the performance of the 4Cr5MoSiV base metal. Besides, fine, and dense α-Al_2_O_3_ was preferentially generated on the surfaces of FeMnCrNiCoAl_x_ (x = 0.5, 0.75) cladding layers. α-Fe_2_O_3_ and Cr_2_O_3_ promoted the heterogeneous nucleation of α-Al_2_O_3_ which effectively improved the high temperature oxidation resistance of the cladding layer. Large, loose (Cr, Fe)_2_O_3_ oxide was formed on the surface of the 4Cr5MoSiV substrate, which deteriorated its high-temperature oxidation resistance. The authors expounded that the combined effects of fine grain strengthening, the difference in lattice structure between FCC and BCC, and dispersion strengthening of rigid BCC phase improved the wear resistance of the FeMnCrNiCoAl_x_ cladding layers at room temperature. Recently, many other researchers published promising results of various HECs developed by cladding, for more information authors suggested to refer those articles [67,173].

## 6. Role of Surface (In Situ) Oxides and Interfacial Processes at Tribological Interface

**Figure 27:**
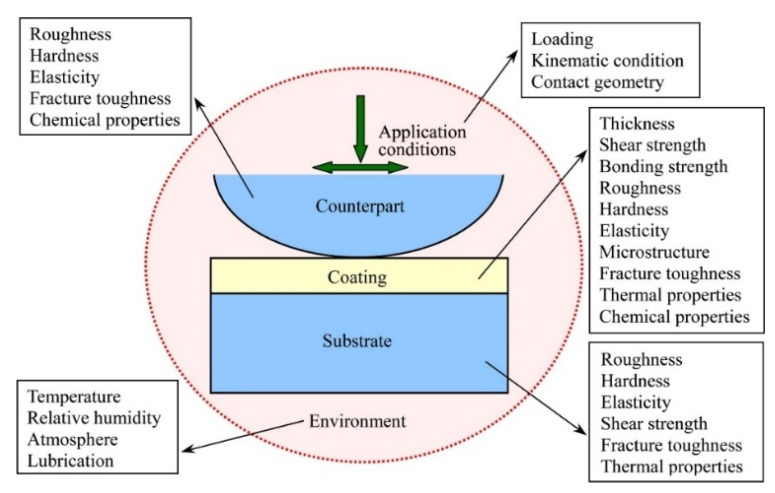
Parameters of a coating system influencing the tribological performance [176].

The demand for mechanical systems that can operate under extreme conditions, such as high loads, speeds, and temperatures, has increased over the last decade. Aerospace systems, advanced combustion engines, mining, and metalworking processes are just a few examples of applications that need the use of high temperatures. The line between low and high temperature is rather vague and ultimately depends on the materials and applications involved. While a temperature of 2000 °C is already above the melting or decomposition point for most metallic alloys and polymers, many ceramics can still provide relatively good mechanical properties above that threshold [175]. In tribology, high temperature applications are those in which conventional lubricants, such as oils and greases, are no longer effective, since they decompose rapidly at around 300 °C. Effect of various parameters on tribological performance: Tribological performance of a coating is not one of its intrinsic properties, but depends on the whole system, including parameters from counterpart, coating, substrate, application conditions and environment as shown in Figure 27.

**Figure 28:**
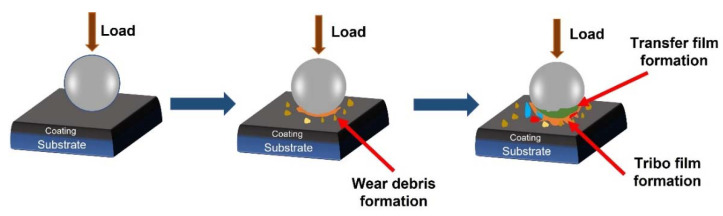
Schematic diagram of tribofilm formation on the wear track under reciprocating sliding at high temperatures.

Mechanical properties such as hardness and yield strength of most metals and alloys decrease with increasing temperatures, with the exception of nickel aluminide alloys (Ni_3_Al) [2,177]. Based on this reduction in inherent mechanical properties, it is fair to expect lower wear resistance at high temperatures. However, this connection should be used with vigilance, because other factors such as microstructural changes, heat conduction, thermal fatigue, material transfer, element segregation forming brittle phases on the substrate, or oxidation can alter the tribo-contact region and thus severely impact the tribological behaviour. Oxide layers form in almost all metals as a result of exposure to oxygen in the air. The interaction of oxygen and metal ions governs the rate of oxidation, which is controlled by temperature. At higher temperatures, chemical reaction rates generally increase. Quinn et al. [178] proposed a general theory of oxidational wear that took into account not just the oxidation induced by frictional heating through interfacial contacts, but also the oxidation that occurs when the sliding surfaces are exposed to ambient temperature [179,180]. The authors revealed that the tribological activation energy for oxidation is roughly half that of static oxidation. In other words, oxidation can occur more easily under sliding conditions than static oxidation subjected to the same ambient temperature. This could be attributable to a higher ion diffusion rate through a developing oxide layer, which is usually filled with defects caused by mechanical perturbations [178,180]. It is also worth noting that the formation of oxide layers during the relative motion of two materials is dependent not only on natural oxides but also on in situ formed ones. This oxide layer formation is often referred to as tribo film (layer) formation as demonstrated in Figure 28.

**Figure 29:**
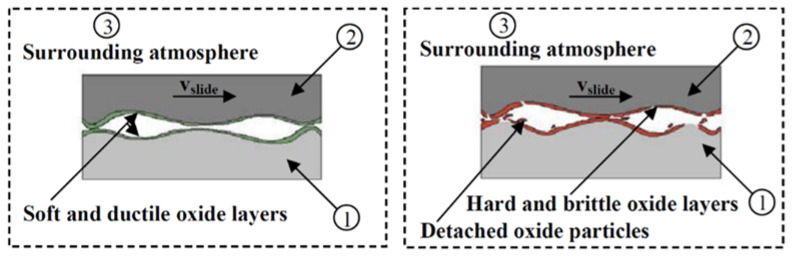
Schematic showing different types of tribo-chemical layers, where (1) High-entropy coatings, (2) Counter surface and (3) Surrounding atmosphere [182].

Stott et al. [181] discovered that three different models for the formation of oxides can occur depending on the sliding and surrounding conditions. The oxidation-scrape-reoxidation process is divided into two stages. Due to the increased local temperature, a general oxidation of the visible contact area as well as the contact asperities occurs at first. This oxide is removed in the second stage by sliding, exposing clean metal for further oxidation. Total oxidation happens at higher ambient temperatures because it involves the formation of oxides during or even before sliding. In this situation, repeated sliding may not completely remove the oxide, allowing the remaining oxide to thicken over time. As seen in Figure 29, the formed oxide layers can be hard and brittle, causing them to detach from the surface and increase friction and wear. On the other hand, the layers can be soft-ductile and lubricious, avoiding direct contact with the substrate and, as a result, decreasing friction.

**Figure 30:**
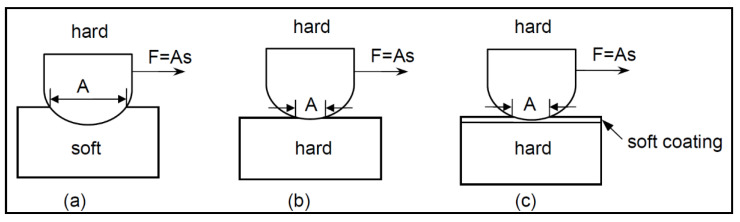
Relation of friction force (F=A.S) to substrate material hardness. (**a**) Hard counterface metal in contact with soft substrate metal (large A and small S), (**b**) similar hardness of counterface metal and substrate metal in contact with each other (small A and large S), (**c**) similar hardness of counterface metal and substrate metal separated by a thin film of soft metal deposited on the substrate surface (both A and S small), where A = contact area and S = shear strength [187].

Stott et al. [183,184,185] described in more depth how the formation of this oxide debris might influence wear behaviour in several ways in subsequent studies. Some particles can be removed completely from the sliding contact and have no further impact on the wear behaviour, while others can be retained inside the wear tracks that move between the sliding surfaces and act as three body abrasives. Finally, these particles can become entrapped and agglomerated in specific regions, particularly grooves, generating compacted layers that operate as load-bearing zones while limiting metal-to-metal contact. Following this process, one of two competing phenomena might occur: the breaking of the layers, resulting in the formation of more debris, or their consolidation through sintering. At higher temperatures, the latter process becomes more favourable. If the oxide becomes solid before it fractures, a glazing layer is formed, and wear is usually reduced to very low levels. However, the presence of oxides at the tribo-contact interface is not always advantageous in terms of wear and friction behaviour. They might have either a beneficial or a negative impact. Detachment of a hard and brittle oxide, for example, may result in increased wear through abrasive particles, whereas detachment of a ductile and lubricious oxide may result in reduced friction and wear. The characteristics of oxides, such as strength, adhesion to the substrate, and capacity to be sintered and therefore form glaze layers, among others, are strongly influenced by the materials in contact as well as the sliding and surrounding conditions. Several researchers [186,187] correlated the friction coefficient to material hardness. For example, hard material sliding over hard surfaces has a high shear strength and consequently a high friction value. Similarly, a hard material rubbing up against a soft one. Since most oxide glaze layers formed at high temperatures exhibit softening and low shear strength, a hard on a soft combination (Figure 30a) is used to improve the tribological performance of tribo materials [187]. Furthermore, the combination pattern of the counter surface and the nature of the coating determine the wear mechanisms at high temperatures [188]. 

Aside from the formation of in situ oxides during sliding, other characteristics of coating/films play a vital role for improved wear resistance. In particular, higher hardness is more resistant to plastic deformation under certain applied loads. According to Leyland [189], the tribological properties of coatings could be evaluated using the values H/E and H^3^/E^2^ (H and E represent hardness and elastic modulus, respectively), which depicted the long elastic strain to failure and the contact yield pressure in a rigid ball on the elastic/plastic plate contact condition, respectively. As a result, increased hardness can contribute to the attractive wear resistance of HEFs and HECs. Apart from the hardness of the tribo-couples, thermal conductivity is an important factor in tribological performance. Higher thermal conductivity causes more heat dissipation to the surrounding regions, resulting in a constant temperature on the contact surface [190,191]. This phenomenon decreases the chance of the contact zone overheating and, as a result, protects the coating from excessive wear. Most ceramics and oxides have low heat conductivity in comparison to metals, resulting in high thermal stresses in the actual contact region. Therefore, HE coatings could be a potential candidate for high-temperature aerospace applications owing to their superior combination of mechanical and thermal properties. However, it is worth noting that there are many different types of HEA systems, and not all of them are suitable for high temperature applications. These different property modifications must be balanced while developing HEA systems for specific purposes by manipulating any of the elements in HEA system.

## 7. Influence of Phase Transition on the Tribological Properties

**Figure 31:**
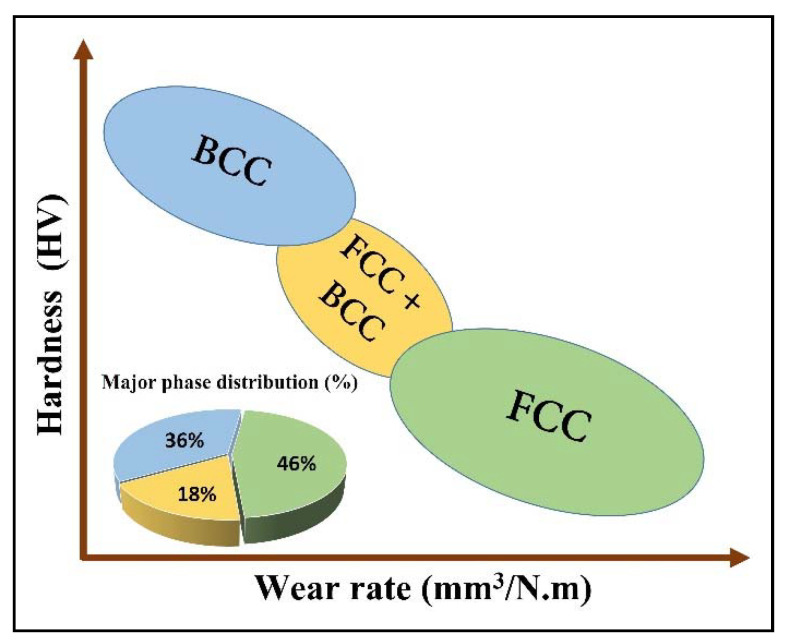
Correlation between hardness and wear rate of HECs categorized based on phase distribution and the phase distribution (%) in various HEA systems. It should be noted that this is a simplified trend and does not include any influence of interfacial processes (e.g., tribo- and transfer-film formation) on the wear behaviour.

According to the literature, the FCC, BCC, or FCC + BCC solid solution phase is most common in HEA systems. Figure 31 depicts the distribution of phases in several HEAs systems. The most common phase type (46%) was FCC, which was also detected in the Cantor alloy (CrMnFeCoNi) and many of its variations. Some studies looked about what ratios of a base element, or an extra element might cause the high-entropy alloy’s main phase to transition from FCC to BCC (18%). Many research studies have also revealed that after the post-heat treatment, the FCC phase transitioned to the BCC phase. In several HEA systems, the distribution of BCC phase was found to be around 36%. Figure 31 illustrates the general trend of hardness vs. wear rate, indicating that the wear resistance increased with the increase in hardness which directly corelates with Archard’s equation (Equation (7)).

(7) $$Q=\frac{K.W.L}{H}$$

where *Q* is the total volume of wear, *K* is a dimensionless constant, *W* is the normal load, *L* is the sliding distance, *H* is the hardness of the softest contacting surfaces.

It should be noted that this is a simplified trend and does not include any influence of interfacial processes (e.g., tribo- and transfer-film formation) on the tribological behaviour. Kasar et al. [192] found a similar trend while reviewing the dry sliding tribological parameters of ingot cast HEAs. As these are the most common crystalline phases in ingot cast HEAs, this article categorises HEA systems based on their crystal structures, FCC, BCC, and FCC + BCC phases.

FCC is a typical phase for HEAs. FCC phases dominate the Cantor alloy, CrMnFeCoNi HEA system [192,193]. Many of the research included in this study focus on what elements need to be added or altered to promote a transition from FCC to BCC phase. According to Guo’s valence electron consideration theory, simple FCC phases dominate a material with a VEC greater than 8 [40]. Since atoms in FCC crystals have a greater number of nearest neighbour atoms, there are more slip systems, which reduce hardness and consequently wear properties. Regardless, an increase in slip systems promotes an improvement in ductility for FCC phase dominated HEAs. This alloy group has the lowest general hardness of the three-phase groups. The soft material also has a direct impact on wear rate because more material is removed during the testing, resulting in much larger wear tracks. Better wear properties may be needed in various applications, although the FCC phase may play a role when high ductility is required. Not all HEAs are made up entirely of FCC or BCC phases. Materials having a VEC between 6.87 and 8 exhibit a mixture of FCC and BCC phases, according to Guo’s valence electron concentration hypothesis. For example, increasing the Al content from 0 to 2.0 molar ratio resulted in an increase in the BCC phase in Al_x_CoCrCuFeNi HEAs [194]. The interdendritic structure transferred from FCC to BCC + FCC with an increase in Al concentration in HEAs made by arc melting; however, the dendritic structures totally changed from FCC to BCC. The rise in Al concentration also resulted in the BCC phase’s spinodal decomposition. Higher hardness and wear coefficient were also achieved because of the microstructure modification and BCC phase evolution. BCC is one of the most desired phases among those found in HEAs [195]. The valence electron concentration (VEC) of a material can be used to anticipate which phases will form in a high-entropy alloy. The dominating phase of the high-entropy alloy is BCC [40] for materials with a VEC less than 6.87.

The comparatively limited slip systems of BCC phases contribute to increased wear properties and promote brittleness in a material. Working toward alloys with a BCC main phase is typically seen as favourable for producing HEAs. FCC is the Cantor alloy, or base alloy, on which many HEAs are based. A substantial amount of research has been conducted to determine what ratios of what elements added to this alloy will induce BCC phases. V [196], Al [194,197,198], Fe [199], and Ti [198] have all been shown to promote the development and stability of BCC phases in HEAs. The addition of these elements to the base high-entropy alloy may improve the possibility of making a material with BCC phases, but at the expense of decreasing the material’s ductility. When designing (HEA systems) for specific purposes, these various property changes must be balanced by manipulating either of element in the system.

## 8. Summary 

During the last 18 years, high-entropy alloys gained global attention owing to their superior combination of properties as compared to conventional alloys. In this article, the authors have reviewed the high-entropy coatings (HECs), including the fabrication routes of HEAs as targets, feedstock and coatings, microstructures, and tribological properties of HECs. The paper is organized into several sections to discuss each property individually. The authors have summarized the tribological and mechanical properties of HECs fabricated using various methods from published articles. The main contents of this paper can be concluded as follows:

- The most common fabrication methods for HEAs in the form of rods or ingots (targets) are vacuum arc melting, mechanical alloying (MA) after spark plasma sintering (SPS), and hot drawing for HEA fibre and wire. In contrast, HEA is also employed as a feedstock in thermal spraying and cladding processes. Four synthesis techniques have been reported to prepare HEA feedstocks: mixing, arc melting followed by mechanical milling, mechanical alloying, and gas atomization.
- The authors analysed numerous coating methods for HECs deposition and summarized the articles in which the researchers developed the coating using various methods such as magnetron sputtering, laser cladding, and thermal spraying. There are several advantages to employing magnetron sputtering, including the ability to combine elements for HEAs directly from single or multicomponent targets. Thermal spray and cladding, on the other hand, has the disadvantage of requiring powder and can still result in the typical tortuous microstructure as well as oxidation and phase decomposition (intermetallic phase) challenges. One process can produce thick coatings (i.e., CrMnFeCoNi HEA) with acceptable mechanical properties, while the other can produce thin films (HE carbides and nitrides) with excellent mechanical properties. Thus, the coating technique used is influenced by the final microstructure and required properties.
- The high-entropy coatings developed by vapor deposition-based methods are mainly composed of HE nitride and carbide coatings. The elements such as Zr, Nv, V, Ti, Hf, etc., possess high affinity with nitrogen, which can easily form nitrides using magnetron sputtering. Aside of the elements, the deposition parameters including N_2_ flow and bias voltage, also have significant influence on the phase formation and final microstructure of the HE nitride coatings. Furthermore, HE nitride coatings form nano-sized structures, resulting in considerable improvements in physical and mechanical properties such as exceptional wear and oxidation resistance and thermal stability. Moreover, the compressive residual stress generated by magnetron sputtering (with manipulation of bias) for the HECs can also reduce the crack formation and contribute to the improved wear resistance.
- The HECs developed using thermal spray methods have shown good microstructural stability with excellent mechanical properties. However, the majority of research has been carried on plasma (APS) and HVOF sprayed HECs, and there is relatively limited information available regarding the tribological behaviour of cold sprayed coatings. Currently, the thermal spray process can only generate high entropy metallic and ceramic coatings through the APS process. Because of temperature limitations in the HVOF and cold spray procedures, they cannot be used to develop High-entropy ceramic coatings. Furthermore, the maximum hardness of ~790 HV for HVOF sprayed Al_0.6_TiCrFeCoNi coating was reported [56]. Plasma-sprayed HECs, on the other hand, outperformed both HVOF and cold-sprayed HECs. So far, the plasma sprayed Al_0.2_TiCrFeCo_1.5_Ni_1.5_ [77] with the addition of 5% Ag has shown the lowest wear rate of 8.9 × 10^−6^ mm^3^/N.m at high temperatures. However, due to the limited open-source literature and lack of high temperature wear data, the commercial potential for thermal sprayed HECs in tribological applications must be evaluated further.
- The cladding process is advantageous for developing HECs with low coating dilution and strong metallurgical bonding between substrate and coatings. According to the literature, cladded coatings formed the FCC or BCC matrix as well as small intermetallic phases. Surprisingly, the presence of solid-solution(s) and intermetallic compound(s) in HECs improved tribological behaviour. The ductile solid-solution matrix can help protect the surface against brittle fracture in the combined structures of solid solution(s) and intermetallic compound(s), while the hard intermetallic compound phase can effectively resist abrasive wear and protect the surface against severe plastic deformation. The combined effects of solid-solution(s) and intermetallic compound(s) can significantly improve the wear resistance of HEFs and HECs. It is worth mentioning that the excellent wear resistance of HEFs and HECs cannot be linked to a single factor, but rather to a combination of factors. The investigation of innovative wear-resistant mechanisms for cladded HECs is important, as it is expected to drive the development of new types of wear-resistant HECs.
- According to the literature, HECs have a high hardness due to the formation of hard phases such as BCC, B2, laves, and ordered phases. Many authors have also reported nitride HECs with extremely high hardness greater than ~40 GPa and discussed the effect of N_2_ concentrations on the mechanical properties of nitride HECs. Whereas cladded and thermally sprayed HECs have been reported to have a maximum hardness of ~10 GPa.
- The authors reviewed the results of tribological testing at room temperature as well as at high temperatures. The authors found that most of the HECs possess a higher wear resistance from room temperature to 750 °C. However, the wear mechanism was abrasive at room temperature to 500 °C, and after that, the dominant mechanism was oxidation.
- The tribological properties of coatings/films can be determined by their mechanical properties. Higher hardness, in particular, is more resistant to plastic deformation under certain applied loads. According to Leyland [189], the tribological properties of coatings could be evaluated using the values H/E and H^3^/E^2^ (H and E represent hardness and elastic modulus, respectively), which depicted the long elastic strain to failure and the contact yield pressure in a rigid ball on the elastic/plastic plate contact condition, respectively. As a result, increased hardness can contribute to the attractive wear resistance of HEFs and HECs.
- As previously stated, HECs have been mostly formed of single solid solution FCC, BCC, and FCC + BCC, with the formation of some minor oxides and an intermetallic phase. The authors attempted to correlate the mechanical and tribological properties of HECs with major phase groups (FCC, BCC, and FCC + BCC). HECs with sole BCC phase showed higher hardness and wear properties relative to FCC due to their limited slip systems but were also brittle. Despite having lower wear properties than the BCC group, FCC phase coatings have remained a viable category for some applications, particularly those requiring some ductility. Furthermore, FCC + BCC groups exhibited properties that were intermediate between BCC and FCC groups and may be an interesting group to investigate further with an optimistic future of HECs.

## 9. Future Scope of High-Entropy Coatings (HECs) for Various Applications

Immense research has been done on the various HECs during the last 18 years. Most of the researchers reported the attractive properties of different HECs, such as high hardness, superior wear, corrosion, and oxidation resistance, together with appealing electrical and magnetic properties. In this section, of future work, the authors have tried to explain the future trends of HECs in terms of optimization of process parameters, evaluation of microstructure and various properties.

**Figure 32:**
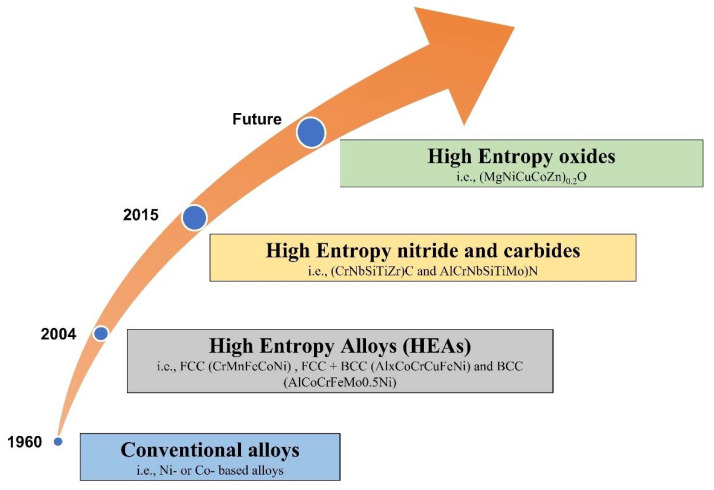
Evolution of high-entropy materials (HEMs) for several applications.

- There is very limited literature available on how to manufacture HEA targets and rods using various manufacturing methods. Hence, it provides a chance to investigate alternate processes in order to get a competitive edge over effective fabrication. According to Kasar et al. [192], one possibility is to use an additive manufacturing technology, such as laser power bed diffusion, to synthesize HEA targets with lubricating elements. These developed HEAs targets/rods with lubricious elements such as Mo, Cu, Nb, Zr, and Ta can be used in the magnetron sputtering method to develop HEFs. However, in order to set the matrix of HEAs compared to conventional alloys, the feasibility of the process and cost factor must be analysed.
- There is very little information available that can reveal the correlation between the powder morphology and the final microstructure and properties of HECs. Therefore, advanced research needs to be carried out on the various HECs by manipulating the powder particle size and morphologies to reveal the relationships between powder particles and microstructure and properties.
- Based on the literature, nitride HE coatings can provide higher mechanical and thermal properties. Also, many authors reported nitride coatings to possess the same properties as superalloys. As suggested by W. Li et al. [33], the effects of the non-nitride-forming element(s) on the microstructures and mechanical properties of HE nitride coatings are still not clear. Therefore, needs further research to understand the strengthening mechanism of the HE nitride coatings and develop the new HE nitride film systems with excellent hardness.
- Based on the design criteria, such as high hardness, the mixture of solid-solutions and intermetallic compounds, the compressive residual stress, and the inclusion of some lubricious elements, new types of HEAs and HECs with the superior wear resistance should be designed, fabricated, and applied.
- This review paper summarizes the different HECs formed by various coating methods. As pointed highlighted by Meghwal et al. [82], there is limited information provided in the literature related to testing conditions and standard practices. Hence, the authors strongly recommend to all the researchers to follow the standard methods for microstructure and thermal characterization [199,200,201,202], tribological testing and mechanical testing procedures [115].
- Currently, more efforts are addressed towards developing HE coatings with improved wear resistance to meet the requirements of industry. In the last decade, the HECs gained global attention due to their combination of properties and novelty. However, there is very limited information available on the efficient means of producing HE carbides and nitrides with higher coating thicknesses. Since the HE carbides and nitrides exhibit excellent mechanical properties, many researchers are optimistic about the future of these HE carbides and nitrides. Figure 32 demonstrates the future trend (in other words evolution) of HEAs. High entropy oxides are also gaining attention of many researchers as a potential solution to these high temperature tribological issues. High-entropy oxides (HEOs) are complex oxides with a single-phase crystal structure and five or more distinct metal cations in the same amount. There have been relatively fewer studies on HEO to date; the first HEO, (MgNiCuCoZn)_0.2_O in a rock salt structure, was reported in 2015 by Rost et al. [203]. According to the authors, the ability to stabilize all these binary compounds into a single rock salt phase is a direct result of the value of the configurational entropy, which is exceptionally large in the case of a multicomponent (generally up to 5 components) mixture and compensates for the unfavourable correspondent enthalpic contribution. However, for this new class of oxide systems to find the optimum composition regime for obtaining desired functional properties, a combination of experiments and theoretical calculations is needed.

A final comment on the future trends is based on the modelling and simulations of the HECs. The mathematical modelling and simulations are beneficial for designing the process parameters and coatings to achieve the desirable properties and microstructures. Therefore, more investigations related to the predictive computational modelling of the HECs need to be explored. Finally, the authors are more optimistic about the HECs, and that many unknown results and mechanisms will be revealed soon.

## Figures and Tables

**Table 1:** Particle size ranges, morphology, and phases present in various high-entropy alloys powder manufactured by different methods.

Powder Preparation Methods	Discussion	Particle Size and Geometry	Phases Observed	Refs.
Blending	Mixing of the powders without promoting any bonding between the particles (Not recommended)	75–80 µm with irregular shape	BCC + FCC	[66,114]
Arc Melting + Mechanical milling	First, intermix the desired alloy by arc melting and then crush the ingot into smaller particles with ball milling (partially recommended due to powder flowability)	30–45 µm with irregular shape	Ordered BCC and FCC	[49,57,116,117]
Mechanical alloying	High-speed rotation and high-energy impacts for cold welding and fracturing, resulting in ‘mixing’ at an atomic scale (recommended)	Wet Milling: 20–35 µm with irregular shape	BCC+ FCC	[64,102,115,118,119,120]
		Dry Milling: 20–70 µm with flaky structure		
Gas atomization	The liquid alloy passes through a nozzle under high pressure (inert gas) and then the fragmentation of liquid streams into spherical droplets	30–220 µm with spherical particles	BCC + B2	[55,104,114,121,122,123,124,125,126,127]

**Table 2:** Summary of various coating methods and characteristics of HECs after each process.

Coating Methods	Classification of Coating Methods	Process Description	Characteristics of Coatings	Refs.
**Vapor deposition and related methods**	**Magnetron Sputtering**	The surface of the target material is eroded by high-energy ions within the confined gaseous plasma, and the liberated atoms travel through the vacuum environment and deposit onto a substrate to form a thin film [38]. Limited for thin-films coatings.	Columnar or epitaxial structure with FCC and/or amorphous solid solution, the thickness can be achieved from 1 µm to 5µ m with a hardness of 7.9 to 10 GPa for high entropy (HE) metallic coatings and (>10 GPa) for HE nitride coatings.	[129,130,131,132,133,134,135,136,137]
	**Vacuum arc deposition**	Deposition of thin film by using the heat energy of arc to evaporate the target materials onto the surface of the substrate [38].	Columnar structure with FCC and BCC solid solution, the thickness can be achieved from 1 µm to 10 µm with hardness ranging from 300 to 750 HV, mostly for HE metallic coatings_._	[138,139,140,141]
**Thermal Spraying**	**High-Velocity Oxygen Fuel (HVOF)**	By using the combustion heat of the fuel and oxygen, spraying the powder particles on the substrate with high velocity. Known for dense coatings with low porosity, low oxide contents, and high bonding Strength [38].	Lamellar grains with BCC solid solution and thickness can be achieved from 100 to 500 µm with a hardness of 700–800 HV, mostly for HE metallic coatings.	[66,70,138]
	**Atmospheric Plasma Spraying (APS)**	Melting of the powder particles by using plasma heat energy source and then spraying the powder particles on the base material. Known for high strength of interfacial bonding, high deposition efficiency and high oxide contents [38].	Lamellar structure with BCC and FCC phase, the thickness of the coating can be achieved from 275 to 570 µm with a hardness of 310–850 HV, mostly for HE metallic coatings.	[41,49,64,66,67,77,117,121,142]
	**Cold Spraying**	Solid-state coating deposition technique, no oxidation, phase transformation and residual thermal stress occur. Limited only for low strength materials [38].	Equiaxed structure with FCC solid solution, the thickness can be achieved from 1 mm to 5 mm with a hardness of 400–550 HV, mostly for HE metallic coatings.	[121,123,142]
**Cladding**	**Laser Cladding**	Pre-placed powders and the thin substrate surface layer are simultaneously melted and solidified rapidly under the heat source of laser [38].	Dendrite or equiaxed grains, BCC or FCC the solid solution with intermetallic compounds and the thickness of 200 µm to 1.5 mm with harness ranging from 345 to 1100 HV, mostly for HE metallic coatings.	[54,60,89,90,99,100,143,144,145,146,147,148,149,150]
	**Plasma Cladding**	Melting with higher heat input and bigger blowing force and mixing of the molten powder are abundant to obtain the homogeneous coating [38].	Columnar or equiaxed grains, BCC or FCC solid solution and the thickness can be produced from (1 mm to 2.5 mm) with harness ranging from 485 to 730 HV, mostly for HE metallic coatings.	[76,151,152,153]

**Table 3:** Volume wear rate of vapor deposition methods, thermal sprayed and cladded high-entropy coatings (HECs). (As some of the wear tests were conducted using different tools/parameters; these results may not be comparable, but they are useful in understanding the tribological performance of various HECs). **Acronyms:** HiPIMS: High power impulse magnetron sputtering, APS: Atmospheric plasma spray, HVOF: High velocity oxyfuel.

High-Entropy Coating (HEC) System	Deposition Method	Counter Ball	Applied Load (N)	Wear Speed (m/s)	Test Temp. (°C)	Sliding Distance (m)	Volume Wear Rate (×10^−6^ mm^3^/N.m)	Ref.
CuMoTaWV	Spark Plasma Sintering	Si_3_N_4_	5	0.1	RT	200	2.19	[155]
					200		4.94	
					400		2.46	
					600		1.39	
CuMoTaWV	Magnetron sputtering	E52100 steel	1	0.1	RT	50	6.4	[156]
					300		25	
AlCrTiVSi	Magnetron sputtering	GCr15 steel	1	0.0157	RT	28	Unmeasurable	[157]
AlCrTiVSi-N							Unmeasurable	
AlCrTiVSi	Magnetron sputtering	Al_2_O_3_	1	0.0157	RT	28	Wear out	
AlCrTiVSi-N							21 ± 2.4	
TiTaHfNbZr	Magnetron sputtering	Al_2_O_3_	1	0.01	RT	30	230	[130]
			2				660	
			3				630	
AlCrNbSiTiMo) N	Magnetron sputtering	Al_2_O_3_	3	0.032	700	75	1.2	[79]
(CrNbSiTiZr)C	Magnetron sputtering	GCr15 steel	2	0.12	RT	216	4.2	[161]
(AlCrTiVZr)N	HiPIMS	GCr15 steel	30	0.004	RT	29	0.23	[160]
AlSiTiCrFeCoNiMo_0.5_	APS	Al_2_O_3_	10	0.5	200	20	4.94	[49]
AlSiTiCrFeNiMo_0.5_	APS	Al_2_O_3_	10	0.5	400	20	2.46	
AlSiTiCrFeNiMo_0.5_	APS	Al_2_O_3_	10	0.5	600	20	1.39	
AlCoCrFeNiSi	APS	Si_3_N_4_	5	0.3	RT	540	38 ± 8	[121]
AlCoCrFeNiTi	APS	Si_3_N_4_	5	0.3	500	360	93 ± 10	[64]
					700		23 ± 10	
					900		43 ± 20	
AlSi_0.2_Ti_0.2_CrFe_0.2_Co_0.6_Ni_0.2_	APS	SiC	10	0.5	RT	20	479 ± 12	[117]
	HVOF	SiC	10	0.5		20	509 ± 17	
CrFeCoNiMo_0.2_	APS	GCr15 steel	10	0.05	RT	45	3.9	[41]
CrFeCoNiMo_0.2_	HVOF	GCr15 steel	10	0.05		45	480	
AlTiCrFeCoNi/Ni_60_	APS	Si_3_N_4_	5	0.3	RT	360	55 ± 6	[66]
					500		67 ± 5	
Al_0.2_TiCrFeCo_1.5_Ni_1.5_-5 wt.% Ag	APS	Si_3_N_4_	5	0.157	RT	566	8	[77]
					200		19.7	
					400		52.6	
					600		52.8	
					750		4.8	
Al_0.2_TiCrFeCo_1.5_Ni_1.5_	APS	Si_3_N_4_	5	0.157	RT	566	25	[77]
					200		31	
					400		81.7	
					600		124.4	
					750		48	
Al_0.5_SiCrFeCoNi	APS	WC-12Co	20	0.04	RT	100	55	[142]
Al_1.0_SiCrFeCoNi							43	
Al_1.5_SiCrFeCoNi							30	
CrMnFeCoNi	APS	WC-Co	20	---	RT	100	270	[67]
CrMnFeCoNi	HVOF	Al_2_O_3_	5	0.0314	RT	100	393 ± 32	[81]
CrMnFeCoNi-annealed							365 ± 41	
Al_0.6_TiCrFeCoNi	HVOF	Al_2_O_3_	5	0.10	RT	500	104.4	[56]
					300		275.7	
					500		267.4	
CrMnFeCoNi	Cold spray	WC-Co	5	0.1	RT	200	476 ± 22	[104]
CoCrFeNiTi_0.5_	Laser cladding	WC	10	----	RT	216	41.68	[174]
					250		50.92	
					500		92.59	
CoCrFeNiTi_0.5_Al_0.5_	Laser cladding	WC	10	----	RT	216	42.12	[174]
					250		45.83	
					500		57.87	
CoCrFeNiTi_0.5_Al	Laser cladding	WC	10	----	RT	216	2.3	[174]
					250		41.87	
					500		50.14	
TiVCrAlSi	Laser cladding	GCr15 steel	10	----	RT- 5Hz	566	25 ± 2	[59]
					RT-10Hz		22 ± 5	
					RT-15Hz		21 ± 5	
Al_0.8_CrFeCoNiCu_0.5_	Laser cladding	GCr15 steel	98	0.209	RT	360	11.9	[171]
Al_0.8_CrFeCoNiCu_0.75_							14.1	
Al_0.8_CrFeCoNiCu							19.0	
CoCrFeMnNi	Plasma cladding	Si_3_N_4_	25	0.157	RT	188	64.	[76]
					200		39.7	
					400		22.5	
					600		36.0	
					800		38.0	
CoCrFeMnNi)_85_Ti_15_	Plasma cladding	Si_3_N_4_	25	0.157	RT	188	25.0	[76]
					200		17.0	
					400		4.5	
					600		8.2	
					800		17.8	

## Data Availability

The data presented in this study are available from the corresponding authors upon request.

## Abbreviations

Important acronyms used in this article: High Entropy (HE); High Entropy Alloys (HEAs); High Entropy Materials (HEMs); High Entropy Coatings (HECs); High Entropy Films (HEFs); High En-tropy Oxides (HEOs).
